# Hypertriglyceridemia in *Apoa5^–/–^* mice results from reduced amounts of lipoprotein lipase in the capillary lumen

**DOI:** 10.1172/JCI172600

**Published:** 2023-12-01

**Authors:** Ye Yang, Anne P. Beigneux, Wenxin Song, Le Phuong Nguyen, Hyesoo Jung, Yiping Tu, Thomas A. Weston, Caitlyn M. Tran, Katherine Xie, Rachel G. Yu, Anh P. Tran, Kazuya Miyashita, Katsuyuki Nakajima, Masami Murakami, Yan Q. Chen, Eugene Y. Zhen, Joonyoung R. Kim, Paul H. Kim, Gabriel Birrane, Peter Tontonoz, Michael Ploug, Robert J. Konrad, Loren G. Fong, Stephen G. Young

**Affiliations:** 1 Department of Medicine and; 2 Human Genetics, David Geffen School of Medicine, UCLA, Los Angeles, California, USA.; 3 Department of Clinical Laboratory Medicine, Gunma University, Graduate School of Medicine, Maebashi, Gunma, Japan.; 4 Lilly Research Laboratories, Eli Lilly and Company, Indianapolis, Indiana, USA.; 5 Division of Experimental Medicine, Beth Israel Deaconess Medical Center, Boston, Massachusetts, USA.; 6 Department of Pathology and Laboratory Medicine, UCLA, Los Angeles, California, USA.; 7 Finsen Laboratory, Copenhagen University Hospital–Rigshospitalet, Copenhagen, Denmark.; 8 Biotech Research and Innovation Centre (BRIC), University of Copenhagen, Copenhagen, Denmark.

**Keywords:** Metabolism, Vascular Biology, Endothelial cells, Lipoproteins, Mouse models

## Abstract

Why apolipoprotein AV (APOA5) deficiency causes hypertriglyceridemia has remained unclear, but we have suspected that the underlying cause is reduced amounts of lipoprotein lipase (LPL) in capillaries. By routine immunohistochemistry, we observed reduced LPL staining of heart and brown adipose tissue (BAT) capillaries in *Apoa5^–/–^* mice. Also, after an intravenous injection of LPL-, CD31-, and GPIHBP1-specific mAbs, the binding of LPL Abs to heart and BAT capillaries (relative to CD31 or GPIHBP1 Abs) was reduced in *Apoa5^–/–^* mice. LPL levels in the postheparin plasma were also lower in *Apoa5^–/–^* mice. We suspected that a recent biochemical observation — that APOA5 binds to the ANGPTL3/8 complex and suppresses its capacity to inhibit LPL catalytic activity — could be related to the low intracapillary LPL levels in *Apoa5^–/–^* mice. We showed that an ANGPTL3/8-specific mAb (IBA490) and APOA5 normalized plasma triglyceride (TG) levels and intracapillary LPL levels in *Apoa5^–/–^* mice. We also showed that ANGPTL3/8 detached LPL from heparan sulfate proteoglycans and GPIHBP1 on the surface of cells and that the LPL detachment was blocked by IBA490 and APOA5. Our studies explain the hypertriglyceridemia in *Apoa5^–/–^* mice and further illuminate the molecular mechanisms that regulate plasma TG metabolism.

## Graphical abstract

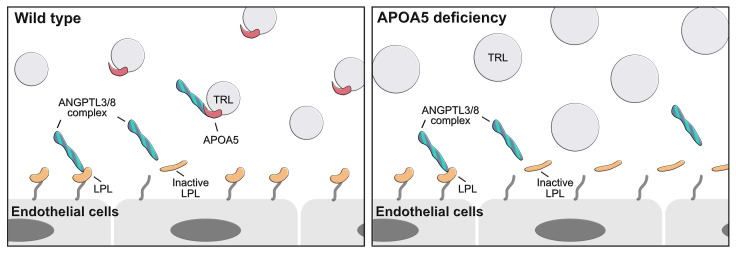

## Introduction

Apolipoprotein AV (APOA5), uncovered by comparative sequencing of the mouse and human *APOAI/CIII/AIV* gene cluster (1), has substantial effects on plasma triglyceride (TG) metabolism. *Apoa5^–/–^* mice have markedly elevated plasma TG levels, with a striking accumulation of large-diameter TG-rich lipoproteins (TRLs) (2, 3). In contrast, overexpression of human *APOA5* lowers plasma TG levels (1). APOA5 is clinically important; *APOA5* mutations result in hypertriglyceridemia (4–9) and an increased risk of coronary heart disease (4, 10, 11).

APOA5 deficiency retards the processing of TRLs (2, 3, 12). For example, the processing and clearance of TRLs are delayed in *Apoa5^–/–^* mice, as judged by studies involving chylomicrons labeled with [^14^C]palmitate and [^3^H]cholesterol (12). Despite considerable effort, however, the mechanisms by which APOA5 deficiency impairs TRL processing have remained unclear. One idea has been that APOA5 interacts directly with lipoprotein lipase (LPL) and activates its TG hydrolase activity (13–15), but several studies did not detect any effect of APOA5 on LPL catalytic activity (16–18). Another idea has been that APOA5, by virtue of its positively charged heparin-binding domain, increases the margination of TRLs along the luminal surface of capillaries by promoting interactions with heparan sulfate proteoglycans (HSPGs) (16, 17). This idea, however, is open to question because the levels of APOA5 in the plasma are extremely low, such that only a small percentage of TRLs contain a single molecule of APOA5 (19–21). Also, the margination of TRLs along capillaries appears to be mediated by LPL on the luminal surface of capillaries (22).

Because LPL is so crucial for TRL processing and because TRL processing is impaired in the setting of APOA5 deficiency, several studies have examined a link between APOA5 deficiency and LPL expression (2, 5–9, 12, 17). Each of these studies has drawn inferences about intravascular LPL levels from an indirect method — measuring levels of TG hydrolase activity in the plasma after a bolus of heparin. The inferences have been remarkably inconsistent. In humans with *APOA5* mutations, 3 research groups found that postheparin LPL activity levels were low (5–7), while 2 groups concluded that the LPL levels were normal (8, 9). Studies of LPL levels in *Apoa5^–/–^* mice also have been inconsistent. In 1 study, LPL activity levels in the postheparin plasma were reported to be low (12), whereas in other studies the LPL activity levels were judged to be normal (2, 17). We suspect that different experimental protocols along with the inherent instability of LPL contributed to the inconsistent findings. In any case, it is unclear whether measurements of LPL levels in the postheparin plasma accurately reflect the amounts of LPL within blood vessels. To our knowledge, no one has examined the impact of APOA5 deficiency on intravascular LPL levels with a direct experimental approach.

Given the severity of the hypertriglyceridemia in *Apoa5^–/–^* mice, we began with a simple hypothesis: that APOA5 deficiency reduces the amounts of LPL within capillaries. From the outset, we knew that our hypothesis would require us to quantify amounts of LPL on the luminal surface of capillaries with a direct experimental approach (23). With the measurements of intravascular LPL levels in *Apoa5^–/–^* mice underway and with support for our hypothesis rapidly accumulating, we were intrigued by biochemical observations from the laboratory of Robert Konrad (18, 24). They found, with biochemical and biophysical methods, that APOA5 binds to the ANGPTL3/8 complex (a physiologic LPL regulator; refs. 25–28) and interferes with the ability of the ANGPTL3/8 complex to bind to LPL. The binding of ANGPTL3/8 reduced LPL’s capacity to hydrolyze TGs. The relevance of the in vitro observations by Konrad’s group to our finding of reduced amounts of LPL in capillaries of *Apoa5^–/–^* mice was not immediately clear; however, we hypothesized that APOA5, by binding to the ANGPTL3/8 complex, modulates intravascular lipolysis by unfolding LPL conformation and thereby regulating the amounts of active LPL in the capillary lumen. We further hypothesized that the ANGPTL3/8 complex functions in vivo by detaching LPL from its intravascular binding sites and that LPL detachment by ANGPTL3/8 is blocked by APOA5. In the current study, we tested these hypotheses.

## Results

### Apoa5-deficient mice.

Plasma TG levels during ad libitum feeding of a chow diet were 4- to 5-fold higher in *Apoa5^–/–^* mice than in *Apoa5^+/+^* mice (Table 1). After an overnight fast, plasma TG levels remained 2- to 5-fold higher in *Apoa5^–/–^* mice (Table 1). As expected, *Apoa5* transcripts were present in the liver of *Apoa5^+/+^* mice but not *Apoa5^–/–^* mice (Supplemental Figure 1A; supplemental material available online with this article; https://doi.org/10.1172/JCI172600DS1). *Lpl* and *Gpihbp1* transcript expression, relative to *Cd31* transcript expression, was not perturbed in *Apoa5^–/–^* mice (Supplemental Figure 1, B and C). Also, *Apoa5* deficiency did not perturb the expression of *Apoc2*, *Apoc3*, *Angptl3*, *Angptl4*, or *Angptl8* (Supplemental Figure 1, D–H). Oligonucleotide primers are listed in Supplemental Table 1.

### Intravascular LPL levels in Apoa5^–/–^ mice.

**Figure 1:**
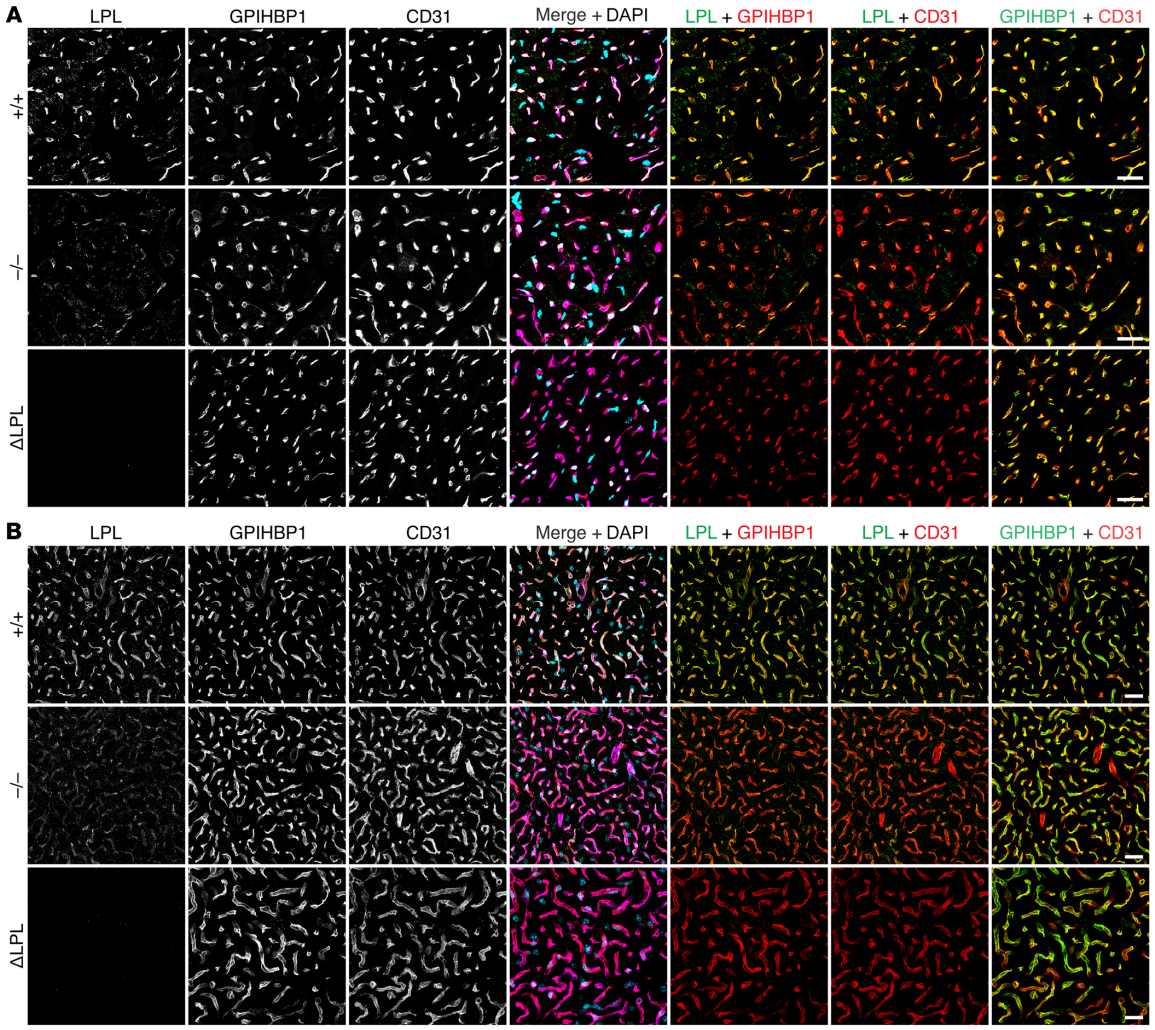
Reduced amounts of mLPL in the heart and BAT of *Apoa5^–/–^* mice. Heart and BAT cryosections from *Apoa5^–/–^* and *Apoa5^+/+^* mice were stained with the mLPL-specific Ab Ab3174, the GPIHBP1-specific mAb 11A12, and the CD31-specific mAb 2H8. Sections from ΔLPL mice, which lack mLPL but express hLPL in endothelial cells, were also examined. (**A** and **B**) Confocal micrographs show mLPL, GPIHBP1, and CD31 staining in heart (**A**) and BAT (**B**). Scale bars: 20 μm.

**Figure 2:**
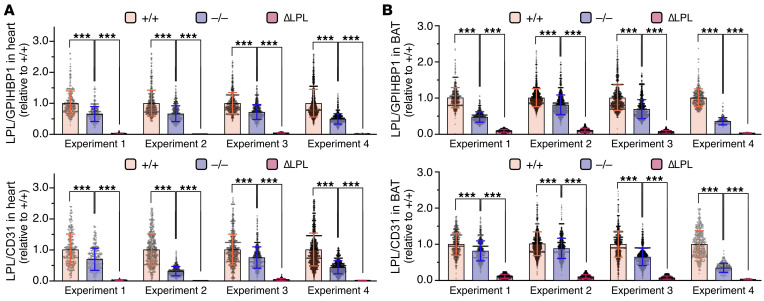
Reduced amounts of mLPL, relative to GPIHBP1 or CD31, in heart and BAT capillaries of *Apoa5^–/–^* mice. Heart and BAT sections from *Apoa5^–/–^*, *Apoa5^+/+^*, and ΔLPL mice were stained with Ab3174, 11A12, and 2H8 (*n* = 4 independent experiments; 2–4 micrographs/tissue section). ΔLPL mice lack mLPL but express hLPL in endothelial cells. Ab3174, 11A12, and 2H8 fluorescence intensities were recorded in individual capillaries; the number of capillaries examined ranged from 323 to 1,870 per group. (**A** and **B**) LPL/GPIHBP1 and LPL/CD31 fluorescence intensity ratios in capillaries of heart (**A**) and BAT (**B**). Each dot represents the signal intensity ratio in a single capillary; ratio data were normalized to the mean ratio in capillaries of *Apoa5^+/+^* mice (set as 1.0). Data represent the mean ± SD. ***P < 0.001, by 1-way ANOVA.

Several studies have drawn inferences about the effect of APOA5 on intravascular LPL levels by measuring LPL activity levels in postheparin plasma, but the inferences have been inconsistent (2, 5–9, 12, 17). We began by using standard immunohistochemical studies to assess LPL levels in tissues of *Apoa5^–/–^* mice. Heart and brown adipose tissue (BAT) sections from *Apoa5^–/–^* and *Apoa5^+/+^* mice were stained with the mouse LPL–specific (mLPL-specific) rabbit polyclonal Ab Ab3174, the GPIHBP1-specific rat mAb 11A12, and the CD31-specific hamster mAb 2H8. (Ab3174 binds preferentially to the N-terminal domain of mLPL; Supplemental Figure 2A.) As a control for Ab specificity, we examined tissues from *Lpl^–/–^* Tie2–hLPL (ΔLPL) mice, in which mLPL is absent. LPL staining, relative to GPIHBP1 and CD31 staining, was reduced in heart and BAT capillaries of *Apoa5^–/–^* mice (Figure 1). In 4 independent experiments, LPL/GPIHBP1 and LPL/CD31 fluorescence intensity ratios in heart capillaries were significantly lower (by 37.2% and 46.0%, respectively) in *Apoa5^–/–^* mice than in *Apoa5^+/+^* mice (Figure 2A). In BAT, the LPL/GPIHBP1 and LPL/CD31 fluorescence intensity ratios were significantly lower (by 42.5 and 33.1%, respectively) in *Apoa5^–/–^* mice (Figure 2B). The GPIHBP1/CD31 fluorescence intensity ratio was not affected by *Apoa5* deficiency (Supplemental Figure 3A).

**Figure 3:**
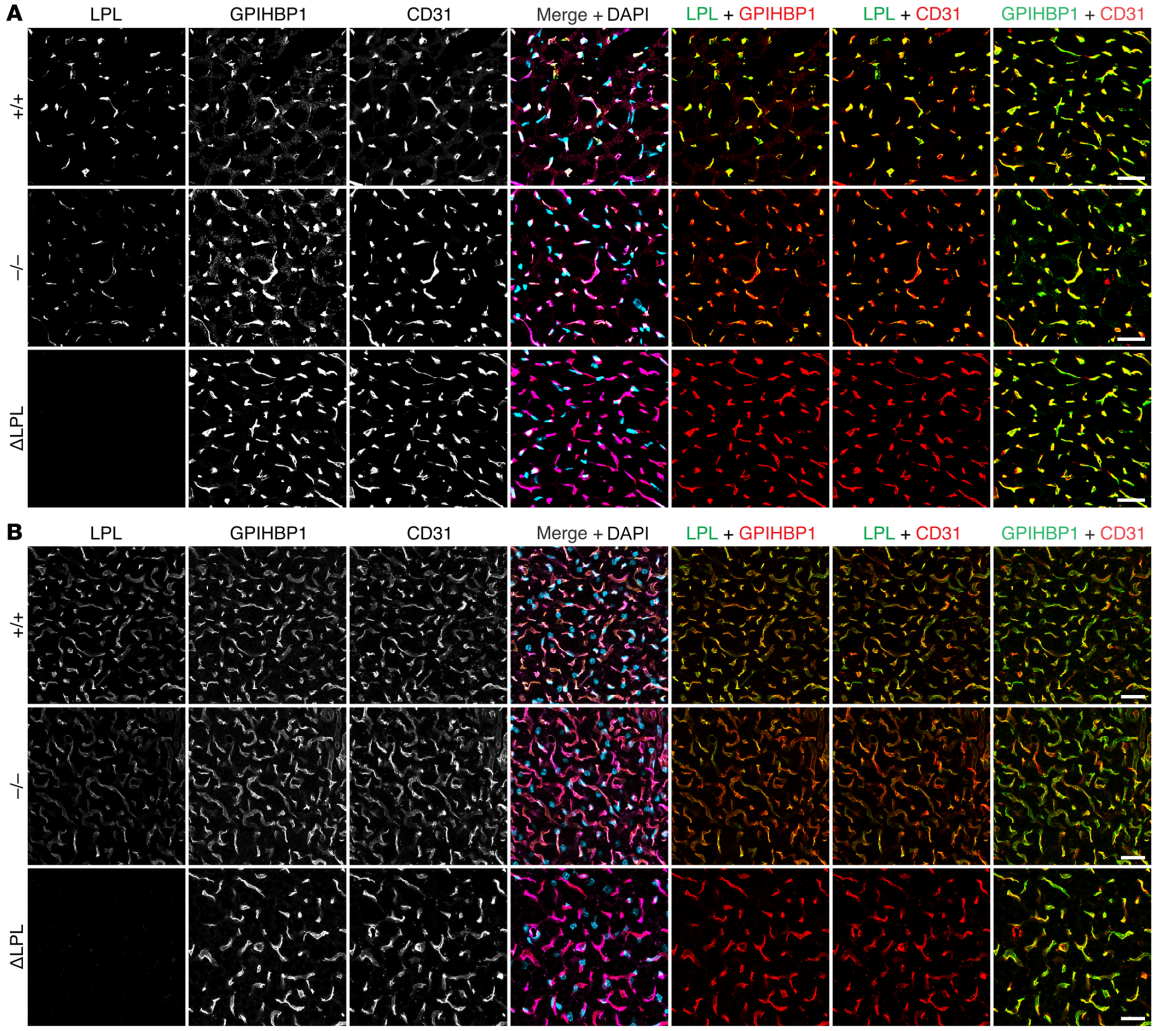
Amounts of LPL along the luminal surface of heart and BAT capillaries, relative to GPIHBP1 or CD31, are lower in *Apoa5*^–/–^ mice. *Apoa5^+/+^*, *Apoa5^–/–^*, and ΔLPL mice were given an intravenous injection of Alexa Fluor–labeled mAbs against mLPL (27A7), GPIHBP1 (11A12), and CD31 (2H8). Ten minutes later, the mice were euthanized; perfused with PBS; and tissue sections were prepared for fluorescence microscopy. (**A** and **B**) Confocal micrographs of LPL, GPIHBP1, and CD31 along the luminal surface of capillaries in heart (**A**) and BAT (**B**). Scale bars: 20 μm.

**Figure 4:**
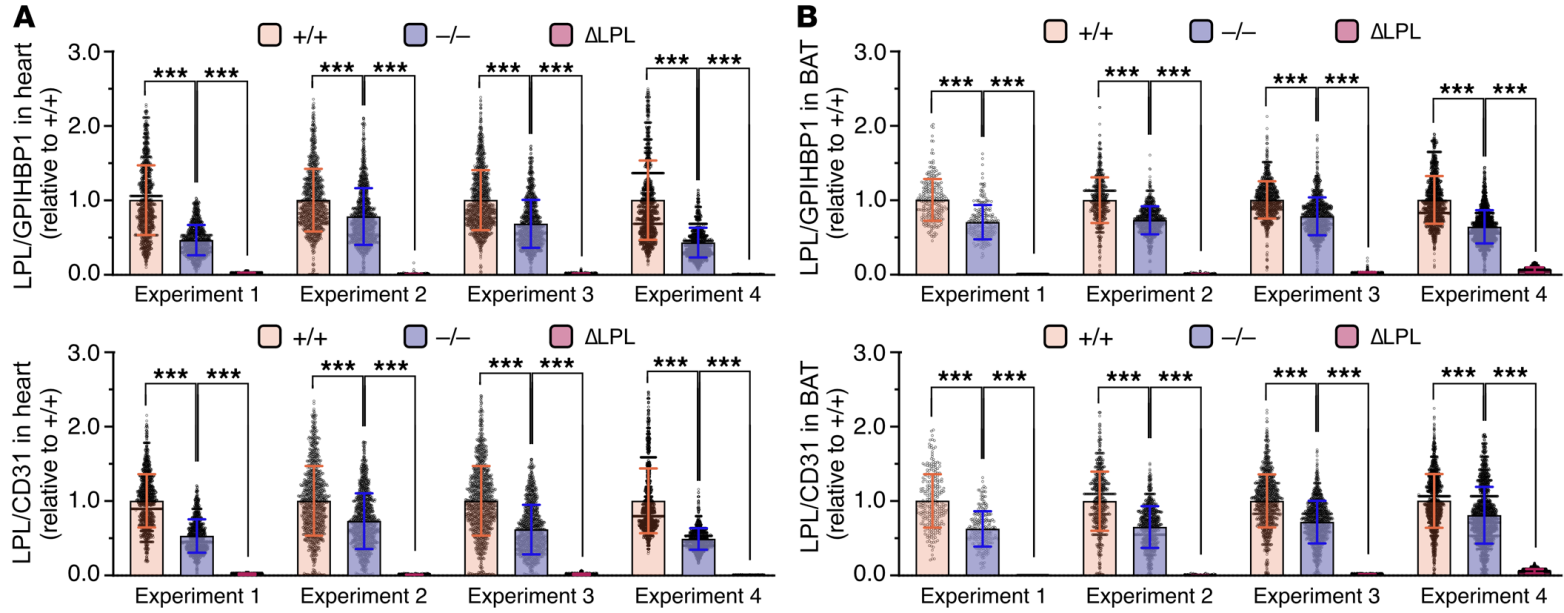
Amounts of LPL in heart and BAT capillaries, relative to GPIHBP1 or CD31, are lower in *Apoa5^–/–^* mice. *Apoa5^–/–^*, *Apoa5^+/+^*, and ΔLPL mice were given an intravenous injection of Alexa Fluor–labeled 27A7 (against mLPL), 11A12 (against GPIHBP1), and 2H8 (against CD31). Following perfusion of the vasculature, tissue sections were prepared for fluorescence microscopy (*n* = 4 independent experiments; 2–4 micrographs/tissue section). 27A7, 11A12, and 2H8 fluorescence intensities in individual capillaries were recorded; the number of heart and BAT capillaries examined ranged from 256 to 2,031. (**A** and **B**) LPL/GPIHBP1 and LPL/CD31 fluorescence intensity ratios in heart (**A**) and BAT (**B**) capillaries of *Apoa5^–/–^*, *Apoa5^+/+^*, and ΔLPL mice. Each dot represents the mean signal intensity ratio in a single capillary, normalized to the mean ratio in capillaries of *Apoa5^+/+^* mice (set as 1.0). Data represent the mean ± SD. ***P < 0.001, by 1-way ANOVA.

While the immunohistochemical studies revealed reduced LPL staining in capillaries of *Apoa5^–/–^* mice, they did not provide definitive insights into the amounts of LPL on the luminal surface of capillaries (where intravascular lipolysis takes place). To address this issue, we gave *Apoa5^–/–^* and *Apoa5^+/+^* mice an intravenous injection of Alex Fluor–labeled mAbs against LPL (27A7), GPIHBP1 (11A12), and CD31 (2H8). (27A7 binds to the C-terminal domain of mLPL, as shown in Supplemental Figure 2B, and binds to LPL•GPIHBP1 complexes, as shown in Supplemental Figure 2C.) Then, after 10 minutes, we prepared tissue sections and assessed, by fluorescence microscopy, Ab binding to the luminal surface of capillaries. The binding of 27A7, relative to 11A12 or 2H8, to the luminal surface of heart and BAT capillaries was lower in *Apoa5^–/–^* mice (Figure 3). In 4 independent experiments, the LPL/GPIHBP1 and LPL/CD31 fluorescence intensity ratios in heart were significantly lower (by 40.9% and 41.1%, respectively) in *Apoa5^–/–^* mice (Figure 4A). In BAT, the LPL/GPIHBP1 and LPL/CD31 ratios were also lower (by 28.9 and 30.0%, respectively) in *Apoa5^–/–^* mice (Figure 4B). The GPIHBP1/CD31 ratios were similar in *Apoa5^–/–^* and *Apoa5^+/+^* mice (Supplemental Figure 3B). In independent studies, we injected Alexa Fluor–labeled Ab3174, 11A12, and 2H8. We found that LPL/GPIHBP1 and LPL/CD31 fluorescence intensity ratios in heart capillaries were, on average, 47.6% and 44.8% lower, respectively, in *Apoa5^–/–^* mice (Supplemental Figure 4).

**Figure 5:**
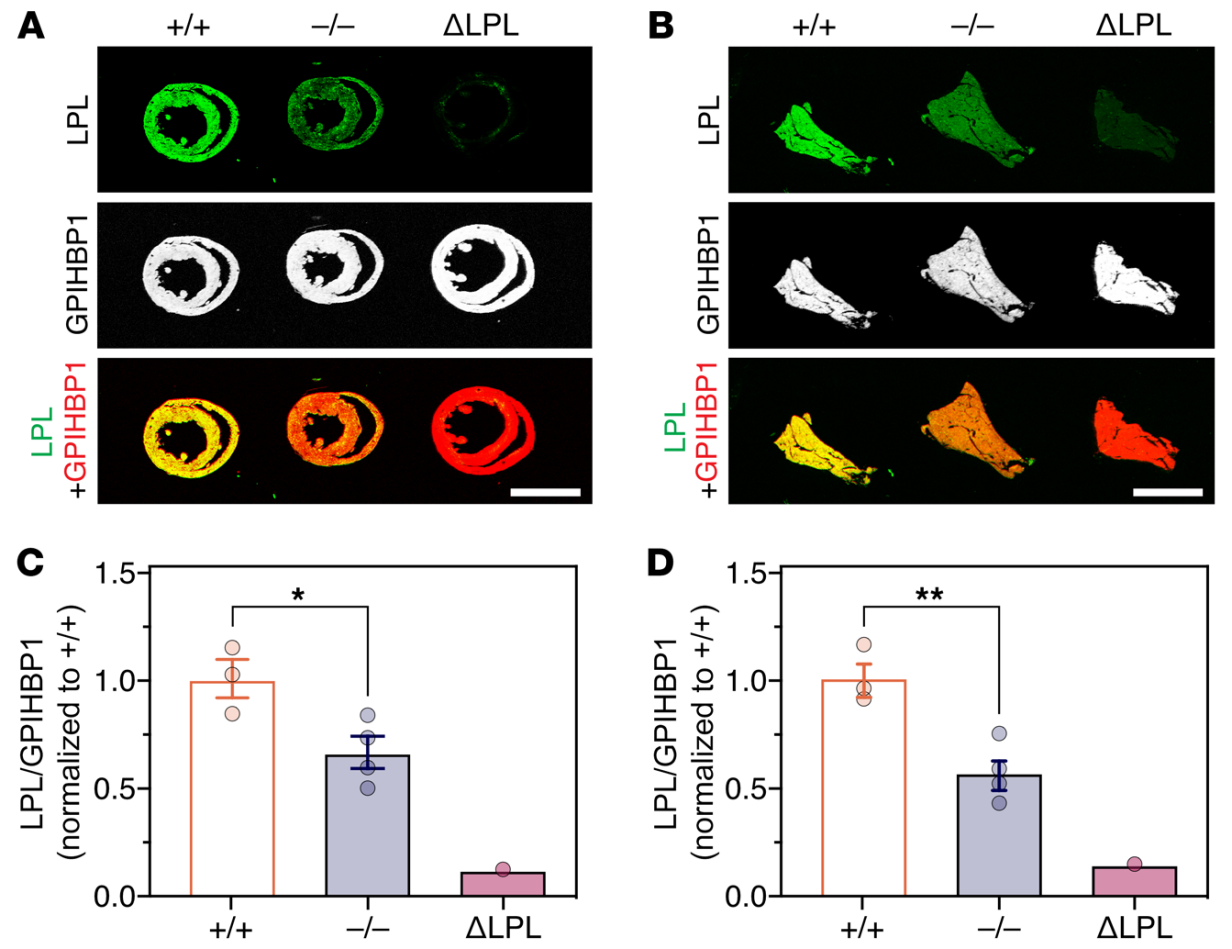
Reduced amounts of LPL in capillaries of *Apoa5^–/–^* mice, as judged by infrared scanning of sections spanning the entire heart or an entire BAT pad. *Apoa5^+/+^*, *Apoa5^–/–^*, and ΔLPL mice were given an intravenous injection of IRDye 680-27A7 and IRDye 800-11A12. Ten minutes later, the mice were euthanized, and the vasculature was perfused with PBS. (**A** and **B**) Infrared scans of heart (**A**) and BAT (**B**) sections revealing reduced amounts of intracapillary LPL, relative to GPIHBP1, in the heart and BAT of *Apoa5^–/–^* mice. Scale bars: 5 mm. (**C** and **D**) LPL/GPIHBP1 ratios in the hearts (**C**) and BAT (**D**) of *Apoa5^–/–^* (*n* = 4) and *Apoa5^+/+^* (*n* = 3) mice. Signal intensities were measured in 10 tissue sections per mouse. Each dot represents the mean LPL/GPIHBP1 signal intensity ratio in 10 sections from 1 mouse; data in *Apoa5^–/–^* and ΔLPL mice were normalized to the mean ratio in *Apoa5^+/+^* mice (set at 1.0). Data show the mean ± SEM. *P < 0.05 and **P < 0.01, by unpaired, 2-tailed Student’s *t* test for differences between *Apoa5^–/–^* and *Apoa5^+/+^* mice.

We also gave mice an intravenous injection of IRDye680-27A7 and IRDye800-11A12 and quantified Ab binding in whole tissue sections with an infrared scanner. Quantification of IRDye signals is more accurate than measuring fluorescence intensity signals with a confocal microscope. The intracapillary binding of 27A7 in *Apoa5^–/–^* mice, relative to that of 11A12, was lower across the entire heart and an entire BAT pad (Figure 5, A and B). The LPL/GPIHBP1 signal intensity ratios in the heart and BAT were significantly lower (by 33.8% and 44.2%, respectively) in *Apoa5^–/–^* mice (Figure 5, C and D). The GPIHBP1/CD31 signal intensity ratios were similar in *Apoa5^–/–^* and *Apoa5^+/+^* mice, as judged by studies involving IRDye680-11A12 and IRDye800-2H8 (Supplemental Figure 3, C–E).

**Figure 6:**
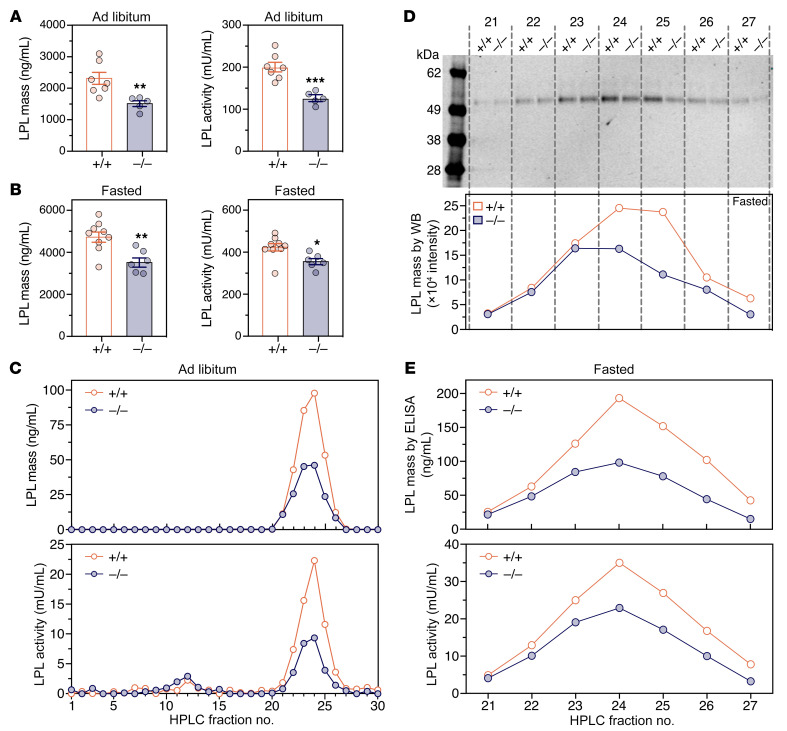
Amounts of LPL in the postheparin plasma are lower in *Apoa5^–/–^* mice than in *Apoa5^+/+^* mice. *Apoa5^–/–^* and *Apoa5^+/+^* mice were given an intravenous injection of heparin (500 U/kg), and plasma samples were collected 2 minutes later. (**A** and **B**) LPL mass and activity levels in the postheparin plasma of individual *Apoa5^–/–^* and *Apoa5^+/+^* mice during ad libitum feeding (**A**) and during fasting (**B**). *Apoa5^–/–^* mice, *n* = 5 in **A** and *n* = 6 in **B**; *Apoa5^+/+^* mice, *n* = 7 in **A** and *n* = 9 in **B**. Data represent the mean ± SEM. *P < 0.05, **P < 0.01, and ***P < 0.001, by unpaired, 2-tailed Student’s *t* test. (**C**–**E**) Amounts of LPL in postheparin plasma from *Apoa5^–/–^* and *Apoa5^+/+^* mice (*n* = 3 mice/group), as assessed by HS chromatography. LPL appeared in the “high-salt” fractions (fractions 21–27; 1.13–1.38 M NaCl). (**C**) LPL mass and activity levels in the HS fractions from the postheparin plasma of *Apoa5^–/–^* and *Apoa5^+/+^* mice during ad libitum feeding. (**D**) Western blots (WB) of LPL in fractions 21–27 from fasted *Apoa5^–/–^* and *Apoa5^+/+^* mice. Band intensity was measured with an infrared scanner. (**E**) Levels of LPL mass and activity in fractions 21–27 from fasted *Apoa5^–/–^* and *Apoa5^+/+^* mice. The reported results were confirmed in 2 independent experiments.

LPL mass and activity levels in the plasma 2 minutes after a bolus of heparin were consistent with the microscopy findings. In mice fed ad libitum, the levels of LPL mass and LPL activity in the postheparin plasma were lower (by 34.3% and 37.0%, respectively) in *Apoa5^–/–^* mice than in *Apoa5^+/+^* mice (Figure 6A). Under fasting conditions, the postheparin LPL mass and activity levels were also lower in *Apoa5^–/–^* mice (by 25.3% and 16.1%, respectively) (Figure 6B). We partially purified LPL from the postheparin plasma by heparin-Sepharose (HS) chromatography and observed that active LPL eluted in the “high-salt” fractions (fractions 21–27) (Figure 6, C–E). In mice fed ad libitum, LPL mass and activity in the high-salt peak was 48.7% and 55.6% lower, respectively, in *Apoa5^–/–^* mice than in *Apoa5^+/+^* mice (Figure 6C). In fasted mice, LPL mass and activity levels in the high-salt peak were also lower (by 33.5% and 45.1%, respectively) in *Apoa5^–/–^* mice (Figure 6, D and E). We also quantified LPL release into the perfusates of isolated mouse hearts after a heparin bolus. In 2 independent experiments, the amounts of LPL released by heparin were lower in *Apoa5^–/–^* mice than in *Apoa5^+/+^* mice (Supplemental Figure 5).

Our studies revealed that the severe hypertriglyceridemia in *Apoa5^–/–^* mice was accompanied by substantial reductions in LPL mass and activity in the postheparin plasma. These observations differed from those in *Lpl^+/–^* mice. Because plasma TG levels in *Lpl^+/–^* mice were only modestly elevated (96.4 mg/dL in *Lpl^+/–^* mice vs. 58.1 mg/dL in *Lpl^+/+^* mice; *n* = 6/group), we anticipated that we would not find substantial differences in LPL levels in the postheparin plasma of *Lpl^+/–^* or *Lpl^+/+^* mice. Indeed, when we measured LPL mass and activity levels in the postheparin plasma of *Lpl^+/–^* and *Lpl^+/+^* mice, we found no significant differences (Supplemental Figure 6). These findings were not surprising. In an earlier study, we could not discern differences in intracapillary LPL levels in the BAT of *Lpl^+/–^* or *Lpl^+/+^* mice (23).

### Assessing the impact of an ANGPTL3/8-specific mAb and recombinant APOA5 in Apoa5^–/–^ mice.

In vitro studies revealed that APOA5 suppresses the ability of the ANGPTL3/8 complex to inhibit the catalytic activity of LPL (18). Our studies revealed reduced amounts of LPL in capillaries in *Apoa5^–/–^* mice and comparably reduced LPL mass and activity measurements, suggesting that unbridled ANGPTL3/8 activity in *Apoa5^–/–^* mice reduced the amounts of catalytically active LPL within capillaries.

**Figure 7:**
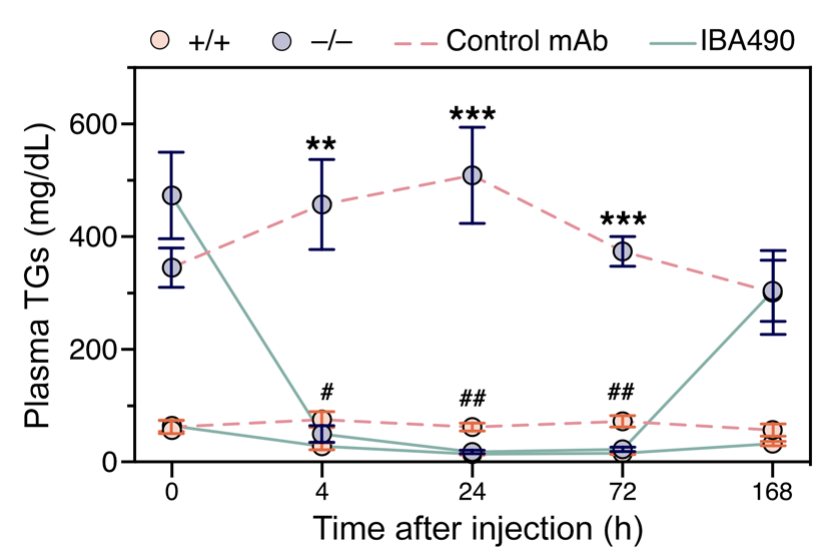
IBA490, an inhibitory ANGPTL3/8-specific mAb, lowers plasma TG levels in *Apoa5^–/–^* mice. *Apoa5^–/–^* and *Apoa5^+/+^* mice were given a subcutaneous injection of IBA490 or a control mAb (10 mg/kg; *n* = 5/group). Plasma samples were obtained at baseline (*t*0) and 4, 24, 72, and 168 hours after administration of the mAbs. Data represent the mean ± SEM. **P < 0.01 and ***P < 0.001, by 2-way ANOVA, for comparisons of plasma TG levels in IBA490- and control mAb–treated *Apoa5^–/–^* mice; ^#^*P* < 0.05 and ^##^*P* < 0.01, for comparisons of TG levels in IBA490- and control mAb–treated *Apoa5^+/+^* mice. There were no significant differences in plasma TG levels in IBA490-treated *Apoa5^–/–^* or IBA490-treated *Apoa5^+/+^* mice at 4, 24, and 72 hours.

To better understand the effect of unsuppressed ANGPTL3/8 activity in *Apoa5^–/–^* mice, we examined the response of *Apoa5^–/–^* mice to an inhibitory ANGPTL3/8-specific mAb, IBA490, which is a chimeric mAb containing the Fc region of mouse IgG and the Fab region of a human mAb that binds to a conformational epitope in the ANGPTL3•ANGPTL8 complex that overlaps with the APOA5 binding site (24). Twenty-four hours after IBA490 administration, plasma TG levels in *Apoa5^–/–^* mice fell from a baseline of approximately 470 mg/dL to approximately 20 mg/dL and remained low for 72 hours (Figure 7). In IBA490-treated *Apoa5^+/+^* mice, plasma TG levels fell from approximately 60 mg/dL to approximately 15 mg/dL (Figure 7).

**Figure 8:**
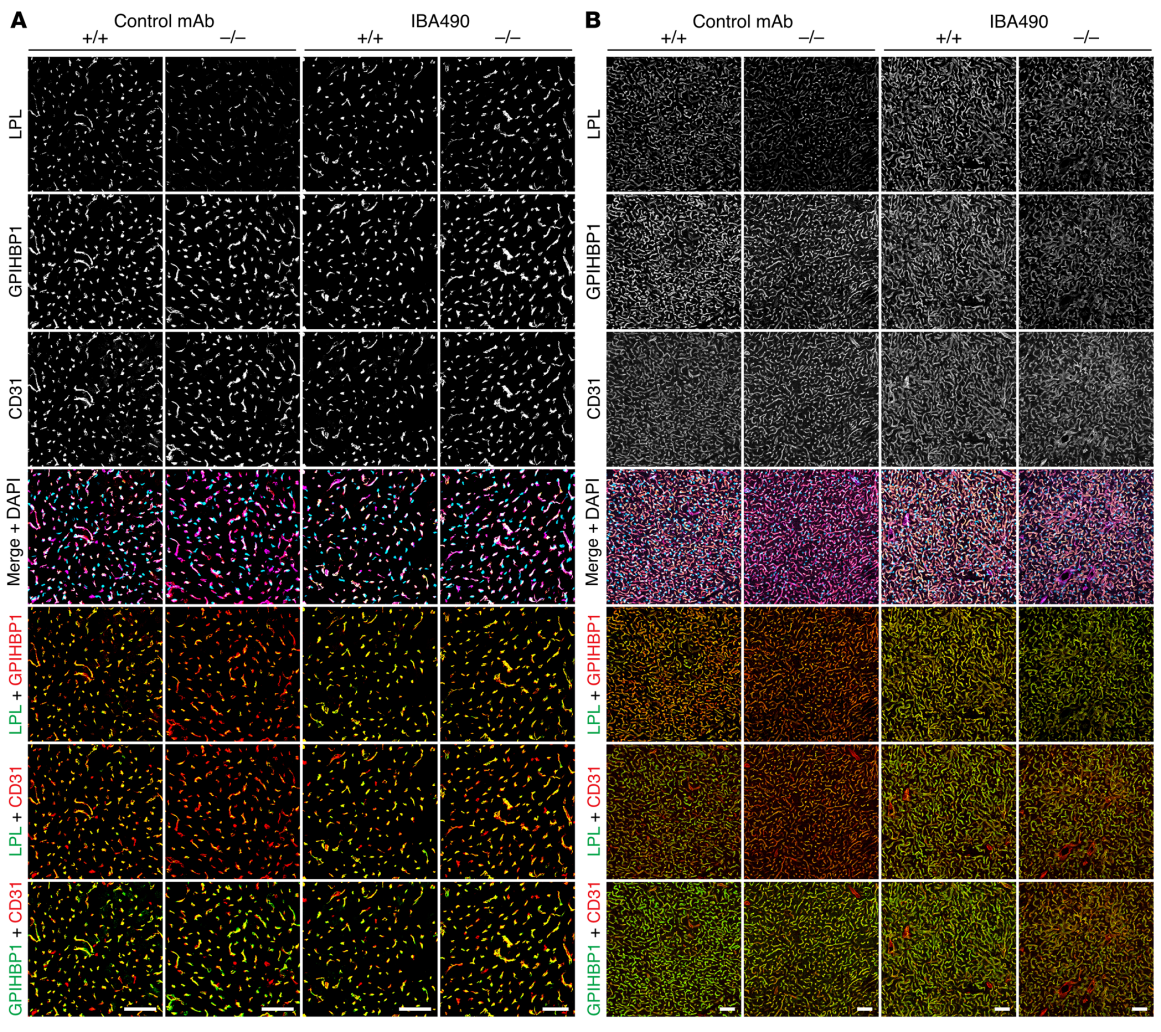
IBA490, an inhibitory ANGPTL3/8-specific mAb, increases intracapillary LPL levels, relative to GPIHBP1 or CD31, in *Apoa5^–/–^* mice. *Apoa5^–/–^* and *Apoa5^+/+^* mice were given a subcutaneous injection of IBA490 or a control mAb (10 mg/kg). Twenty-four hours later, the mice were given an intravenous injection of Alexa Fluor–labeled mAbs against mLPL (27A7), GPIHBP1 (11A12), and CD31 (2H8). After 10 minutes, mice were euthanized and then perfused with PBS, and tissue sections were prepared for fluorescence microscopy. (**A** and **B**) Confocal micrographs of LPL, GPIHBP1, and CD31 staining along the luminal surface of capillaries in heart tissue (**A**) and BAT (**B**) of IBA490- or control mAb–treated *Apoa5^–/–^* and *Apoa5^+/+^* mice. Scale bars: 50 μm. The amounts of intracapillary LPL relative to the amounts of intracapillary GPIHBP1 or CD31 were quantified in 3 independent experiments, and the data are shown in Supplemental Figure 7.

Given that high plasma TG levels in *Apoa5^–/–^* mice were associated with low intracapillary LPL levels, we suspected that the low TG levels in IBA490-treated *Apoa5^–/–^* mice resulted from greater amounts of LPL in capillaries. To test that idea, *Apoa5^–/–^* and *Apoa5^+/+^* mice were given a subcutaneous injection of IBA490 (or an irrelevant control mAb) and, after 24 hours, were administered an intravenous injection of the Alexa Fluor–labeled mAbs 27A7, 11A12, and 2H8. In *Apoa5^–/–^* mice, IBA490 treatment resulted in increased 27A7 binding to capillaries (relative to 11A12 or 2H8), indicating increased amounts of LPL in the capillary lumen (Figure 8). In 3 independent experiments, the LPL/GPIHBP1 and LPL/CD31 fluorescence intensity ratios were significantly higher (by 180.7% and 114.7%, respectively) in heart capillaries of IBA490-treated *Apoa5^–/–^* mice than in the control mAb–treated *Apoa5^–/–^* mice (Supplemental Figure 7A). In BAT, the LPL/GPIHBP1 and LPL/CD31 fluorescence intensity ratios were significantly higher (by 106.9% and 54.5%, respectively) in capillaries of IBA490-treated *Apoa5^–/–^* mice (Supplemental Figure 7B). In the mice treated with a control mAb, the LPL/GPIHBP1 and LPL/CD31 fluorescence intensity ratios in the heart were lower (by 50.4% and 46.3%, respectively) in *Apoa5^–/–^* mice than in *Apoa5^+/+^* mice (Supplemental Figure 7A). In BAT, the LPL/GPIHBP1 and LPL/CD31 fluorescence intensity ratios were lower (by 26.8% and 20.7%, respectively) in *Apoa5^–/–^* mice (Supplemental Figure 7B).

Measurements of LPL mass and activity levels in the postheparin plasma of IBA490-treated mice were consistent with measurements of the amounts of LPL levels within capillaries (as judged by confocal microscopy). Postheparin LPL mass and activity levels were significantly higher (by 58.0% and 55.2%, respectively) in IBA490-treated *Apoa5^–/–^* mice than in control mAb–treated *Apoa5^–/–^* mice (Supplemental Figure 8). As expected, LPL mass and activity levels were significantly lower (by 28.8% and 27.7%, respectively) in control mAb–treated *Apoa5^–/–^* mice than in control mAb–treated *Apoa5^+/+^* mice (Supplemental Figure 8).

In *Gpihbp1^–/–^* mice, in which intravascular LPL levels are negligible (29, 30), IBA490 had no significant effect on postheparin LPL mass levels (368.5 ± 45.4 ng/mL in IBA490-treated mice vs. 353.4 ± 60.3 ng/mL in control mAb–treated mice; mean ± SEM, *n* = 5/group) or postheparin LPL activity levels (12.4 ± 3.0 mU/mL in IBA490-treated mice vs. 29.1 ± 5.8 mU/mL in control mAb–treated mice) (Supplemental Figure 9). In contrast to *Apoa5^–/–^* mice (Figure 7), IBA490 had no significant effects on plasma TG levels in *Gpihbp1^–/–^* mice. In *Gpihbp1^–/–^* mice, plasma TG levels were 1,521.5 ± 220.9 mg/dL at baseline and 1,438.6 ± 249.9 mg/dL 24 hours after IBA490 treatment (mean ± SEM; *n* = 5/group).

**Figure 9:**
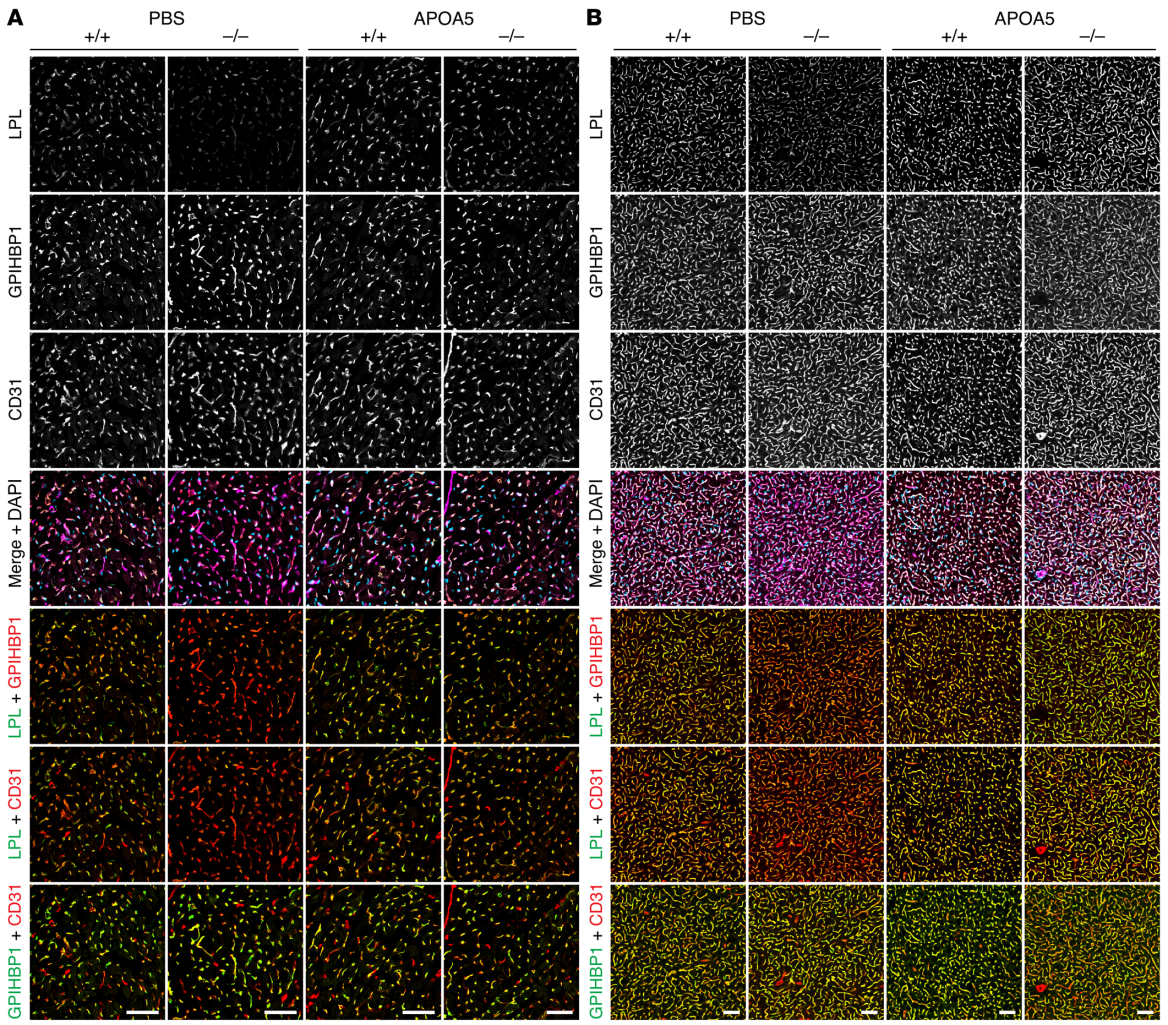
Increased amounts of intracapillary LPL, relative to GPIHBP1 or CD31, in *Apoa5^–/–^* mice after injection of recombinant APOA5. *Apoa5^+/+^* and *Apoa5^–/–^* mice were given an intravenous injection of recombinant APOA5 (10 mg/kg) or PBS alone. After 4 hours, mice were given an intravenous injection of Alexa Fluor–labeled mAbs against LPL (27A7), GPIHBP1 (11A12), and CD31 (2H8). After 10 minutes, the mice were euthanized, the vasculature was perfused with PBS, and tissue sections were examined by fluorescence microscopy. (**A** and **B**) Confocal micrographs of LPL, GPIHBP1, and CD31 staining along the luminal surface of capillaries in the heart (**A**) and BAT (**B**) of *Apoa5^–/–^* and *Apoa5^+/+^* mice that had been treated with APOA5 or PBS alone. Scale bars: 50 μm. The amounts of intracapillary LPL relative to the amounts of intracapillary GPIHBP1 or CD31 were quantified in 3 independent experiments, and data are shown in Supplemental Figure 11.

We suspected that recombinant APOA5 would also lower plasma TG levels and increase intracapillary LPL levels in *Apoa5^–/–^* mice. We gave *Apoa5^–/–^* and *Apoa5^+/+^* mice an intravenous injection of recombinant mouse APOA5 (HIS-MSA-APOA5; 10 mg/kg) or PBS alone. In *Apoa5^–/–^* mice, APOA5 lowered plasma TG levels to below 20 mg/dL, similar to the levels in APOA5-treated *Apoa5^+/+^* mice (Supplemental Figure 10). Lower amounts of APOA5 were also effective; APOA5 (0.1–0.4 mg/kg) reduced plasma TG levels in *Apoa5^–/–^* mice within 4 hours from 605.8 ± 118.2 mg/dL to 64.9 ± 13.6 mg/dL (mean ± SEM; *n* = 6/group; *P* < 0.001). To assess intracapillary LPL levels, APOA5-treated mice were administered an intravenous injection of the Alexa Fluor–labeled mAbs 27A7, 11A12, and 2H8. After 10 minutes, the vasculature was perfused, and sections were prepared for fluorescence microscopy. In 3 independent experiments, the LPL/GPIHBP1 and LPL/CD31 fluorescence intensity ratios in heart capillaries were higher (by 133.9% and 116.1%, respectively) in APOA5-treated *Apoa5^–/–^* mice than in PBS-treated *Apoa5^–/–^* mice (Figure 9A and Supplemental Figure 11A). In BAT capillaries, the LPL/GPIHBP1 and LPL/CD31 fluorescence intensity ratios were significantly higher (by 134.2% and 69.8%, respectively) in APOA5-treated *Apoa5^–/–^* mice (Figure 9B and Supplemental Figure 11B). In mice that received PBS, the LPL/GPIHBP1 and LPL/CD31 fluorescence intensity ratios in heart capillaries were lower (by 37.7% and 43.8%, respectively) in *Apoa5^–/–^* mice (Figure 9A and Supplemental Figure 11A). In BAT, the LPL/GPIHBP1 and LPL/CD31 fluorescence intensity ratios were lower (by 47.4% and 27.8%, respectively) in *Apoa5^–/–^* mice (Figure 9B and Supplemental Figure 11B).

Changes in intravascular LPL activity were consistent with the changes in the amounts of LPL within capillaries. The LPL mass and activity levels in the postheparin plasma were higher (by 60.6% and 65.7%, respectively) in APOA5-treated *Apoa5^–/–^* mice (Supplemental Figure 12). After APOA5 treatment, LPL mass and activity levels in the postheparin plasma of *Apoa5^–/–^* mice were similar to those in *Apoa5^+/+^* mice. In mice that received PBS, the LPL mass and activity levels were lower (by 21.7% and 18.9%, respectively) in *Apoa5^–/–^* mice than in *Apoa5^+/+^* mice (Supplemental Figure 12).

### ANGPTL3/8 releases human LPL from the surface of cells.

**Figure 10:**
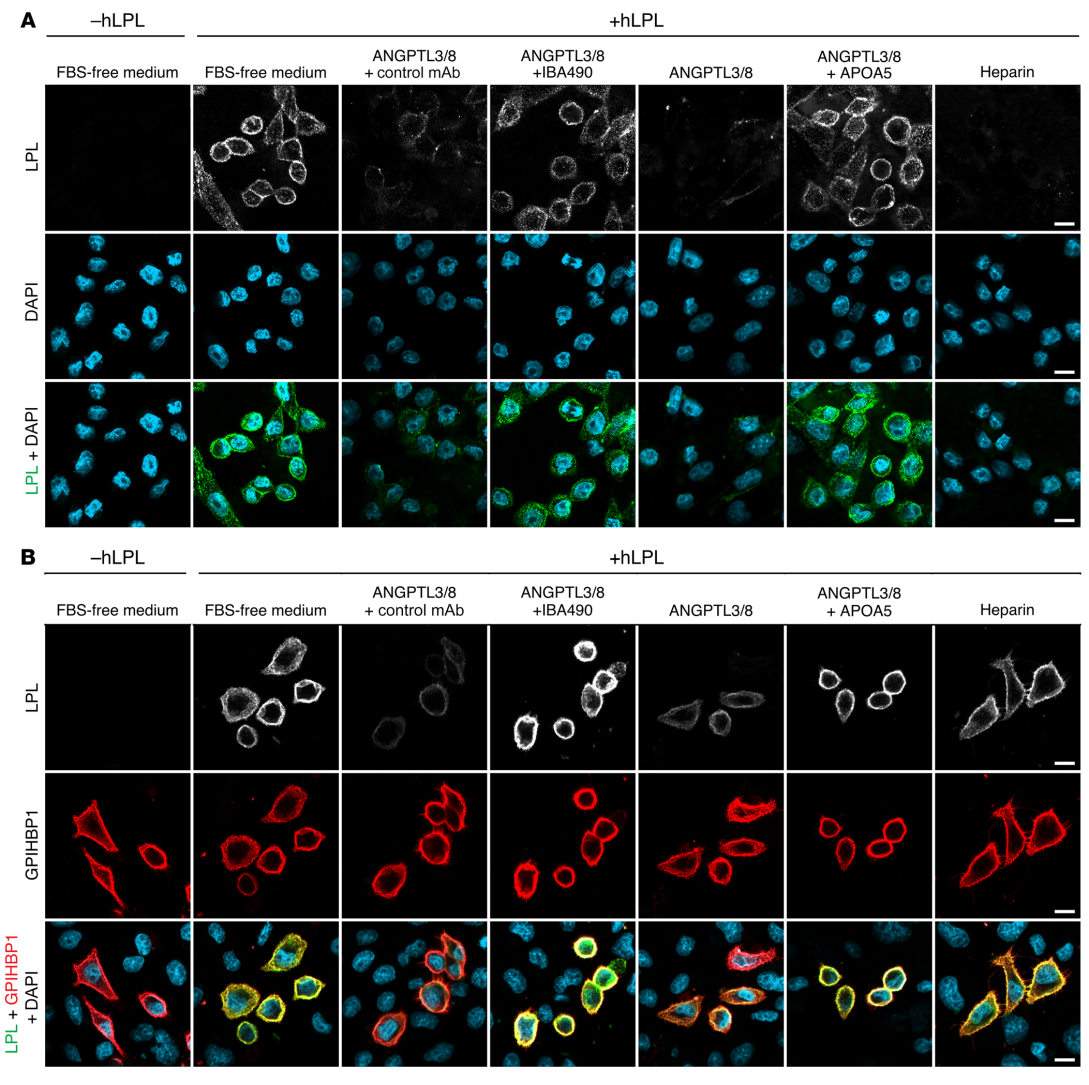
ANGPTL3/8 releases hLPL from the surface of cells. (**A** and **B**) CHO-K1 cells (**A**) and CHO pgsA-745 cells that had been transiently transfected with a mouse GPIHBP1 vector (**B**) were incubated with 50 nM hLPL at 37°C for 10 minutes. After washing the cells with PBS/Ca/Mg, the cells were incubated with 0.1 U/mL heparin or with 100 nM ANGPTL3/8 in the presence or absence of 1 μM control mAb, 1 μM IBA490, or 1.4 μM APOA5 at 37°C for 15 minutes. The cells were washed and cooled on ice for 15 minutes. The amounts of hLPL remaining on the surface of cells were assessed by fluorescence microscopy with an Alexa Fluor–labeled mAb against hLPL (5D2) in **A** or with Alexa Fluor-labeled mAbs 5D2 and 11A12 in **B**. These results were observed in 2 independent experiments. Scale bars: 10 μm.

Intracapillary LPL levels were low in *Apoa5^–/–^* mice but were normalized by IBA490 and APOA5. Those findings suggested that APOA5 deficiency results in unsuppressed ANGPTL3/8 activity, which in turn leads to less LPL on intravascular LPL binding sites. To test whether ANGPTL3/8 is capable of releasing LPL from HSPGs, we loaded the cell-surface HSPGs of CHO-K1 cells (31) with recombinant human LPL (hLPL) by incubating the cells with 50 nM hLPL at 37°C for 10 minutes. After washing, the cells were incubated with heparin (0.1 U/mL) or with recombinant ANGPTL3/8 (100 nM) in the presence or absence of IBA490 (1 μM) or APOA5 (1.4 μM) at 37°C for 15 minutes. Recombinant ANGPTL3/8 was active (18, 24, 32) (Supplemental Figure 13). We then examined, by fluorescence microscopy, the amounts of hLPL (33) on the surface of CHO-K1 cells using the hLPL-specific mAb 5D2 (34). ANGPTL3/8 reduced amounts of LPL on the surface of cells, and the effect was blocked by IBA490 and APOA5 (Figure 10A). Heparin also reduced the amounts of LPL on the cell surface (Figure 10A). Quantification of hLPL release from CHO cells is provided in Supplemental Figure 14A.

We also examined, by fluorescence microscopy, the ability of ANGPTL3/8 to release recombinant hLPL from GPIHBP1 on HSPG-deficient CHO pgsA-745 cells that had been transiently transfected with a mGPIHBP1 expression vector (35) (Figure 10B). The GPIHBP1–transfected cells were incubated with hLPL (50 nM) at 37°C for 10 minutes. After washing the cells, they were incubated with heparin or ANGPTL3/8 in the presence or absence of IBA490 or APOA5. The amount of hLPL on the cells, relative to GPIHBP1, was assessed by fluorescence microscopy with the Alexa Fluor–labeled mAbs 11A12 and 5D2. ANGPTL3/8 released hLPL from GPIHBP1 on the GPIHBP1-transfected cells, and that effect was blocked by IBA490 and APOA5 (Figure 10B). Quantification of hLPL release from GPIHBP1 on the GPIHBP1-expressing cells is shown in Supplemental Figure 14B. We observed similar findings in microvascular endothelial cells expressing GPIHBP1. In those cells, ANGPTL3/8 released LPL, and the release was blocked by IBA490 and APOA5 (Supplemental Figure 15).

In independent studies, we tested whether ANGPTL3/8 increased the release of mLPL from HEK293 cells that stably expressed mLPL (18, 24, 32). ANGPTL3/8 increased the amount of mLPL in the medium (Supplemental Figure 16A), and that effect was minimized by IBA490 and APOA5 (Supplemental Figure 16A). Heparin also released mLPL into the medium (Supplemental Figure 16A). As expected, the TG hydrolase activity of the mLPL released by heparin was high, whereas the activity of the mLPL released by ANGPTL3/8 was low (Supplemental Figure 16B).

## Discussion

We hypothesized that the elevated plasma TG levels in the setting of APOA5 deficiency (1, 2, 12) are caused by reduced amounts of LPL in capillaries. In the current studies, we found strong support for this hypothesis. By routine immunohistochemistry, we observed reduced LPL staining of capillaries in the heart and BAT of *Apoa5^–/–^* mice. Also, when we gave mice an intravenous injection of LPL-, GPIHBP1-, and CD31-specific mAbs, we observed reduced binding of the LPL mAb (relative to the CD31 and GPIHBP1 mAbs) on the luminal surface of heart and BAT capillaries in *Apoa5^–/–^* mice. LPL mass and activity levels in the postheparin plasma (and in postheparin perfusates of isolated hearts) were also reduced in *Apoa5^–/–^* mice. In seeking an explanation for these findings, we were inspired by a recent biochemical finding that APOA5 binds to the ANGPTL3/8 complex and suppresses its ability to bind and inhibit LPL’s TG hydrolase activity (18). We suspected that increased ANGPTL3/8 activity in *Apoa5^–/–^* mice might lead to reduced amounts of LPL in capillaries. With that idea in mind, we predicted that high plasma TG levels and low amounts of intracapillary LPL in *Apoa5^–/–^* mice would be reversed by an inhibitory ANGPTL3/8-specific Ab (IBA490). Indeed, we found that IBA490 treatment of *Apoa5^–/–^* mice normalized plasma TG levels, increased the amounts of LPL within capillaries, and increased the levels of LPL in postheparin plasma. Recombinant APOA5 had the same effects. Our studies explained the hypertriglyceridemia in *Apoa5^–/–^* mice and increased our understanding of the mechanisms that regulate plasma TG metabolism.

ANGPTL3/8 functions in an endocrine manner to inhibit LPL activity in oxidative tissues (e.g., heart, skeletal muscle) (25–28), and ANGPTL8 deficiency is accompanied by higher amounts of LPL in postheparin plasma (28). ANGPTL3/8 inhibits LPL activity in the test tube (24, 27, 32, 36), and APOA5 deficiency would be expected to increase ANGPTL3/8 activity (18), but a puzzle remained. Why would greater amounts of ANGPTL3/8 activity result in reduced amounts of LPL protein inside capillaries? Our earlier studies of a related LPL inhibitor, ANGPTL4, provide important insights (37–40). We showed with hydrogen-deuterium exchange/mass spectrometry studies that ANGPTL4 inhibits LPL activity by catalyzing the unfolding of LPL’s N-terminal catalytic domain (37, 38). ANGPTL4 binds to sequences surrounding LPL’s catalytic pocket (39), triggering a progressive unfolding of the sequences required for the architecture of the hydrolase domain of LPL, including sequences spanning the catalytic triad of LPL (39, 41). The laboratory of Brandon Davies reported that ANGPTL4 prevents LPL binding to GPIHBP1 and that treatment of LPL-GPIHBP1 complexes with ANGPTL4 results in the dissociation of LPL (42). The molecular mechanism by which ANGPTL3/8 inhibits LPL has not yet been defined, to our knowledge, but we know that ANGPTL3/8 binds LPL (24, 27, 36), and we suspect that ANGPTL3/8, like ANGPTL4, promotes LPL unfolding and that the unfolding causes LPL to detach from intracapillary binding sites. In support of that possibility, we found that ANGPTL3/8 released hLPL from the surface of CHO-K1 cells and from GPIHBP1 on GPIHBP1-expressing CHO pgsA-745 cells. Importantly, we also showed the release of LPL from mLPL-expressing HEK-293 cells. In each case, the release of LPL by ANGPTL3/8 was reduced by IBA490 and APOA5. In the future, we hope to use HDX-MS to delineate the binding site of ANGPTL3/8 on LPL and to determine whether that binding event promotes the same LPL-unfolding cascade that is triggered by ANGPTL4 (39, 43).

For years, clinical investigators have drawn inferences about the amounts of intravascular LPL by measuring LPL activity levels (and in some cases LPL mass) in postheparin plasma (5, 6, 8, 9), but this approach has drawbacks. First, measurements of LPL in postheparin plasma cannot provide insights into the origin of the LPL (e.g., whether it is released from adipose tissue or striated muscle). This is an important limitation because LPL is differentially regulated in those tissues (23, 28, 44). Second, whether LPL levels in the postheparin plasma reflect the levels of LPL inside blood vessels or simply reflect the numbers and/or avidity of LPL binding sites has never been clear. Third, the amount of LPL in the postheparin plasma depends on the precise dose of heparin that is injected, the timing of blood sampling, and, presumably, the turnover of heparin in the bloodstream. Fourth, LPL is inherently unstable (39), and the methods for quantifying LPL mass and activity measurements need to be standardized across every laboratory involved in TG metabolism research. We standardized our LPL mass measurements and LPL activity measurements but also applied a new approach. We used mLPL-specific Abs, labeled with either fluorescent or infrared dyes, to assess intracapillary LPL levels. The Ab-based method yielded reproducible results in independent experiments, and we believe that it represents a very useful means of quantifying the amounts of intravascular LPL in tissues (23). It is true that the LPL levels in postheparin plasma from *Apoa5^–/–^* mice were consistent with the Ab-based findings, but at this point it remains unclear whether the 2 methods would invariably yield concordant findings in more complex experimental models. It would be interesting, for example, to compare intracapillary LPL levels and postheparin LPL levels during treatment with PPAR agonists, which affect the expression of dozens of genes, including multiple genes relevant to TRL processing (45, 46).

In our studies, the reduced amounts of LPL activity in the postheparin plasma from *Apoa5^–/–^* mice were accompanied by similar reductions in the amounts of LPL mass in the postheparin plasma. This finding was somewhat surprising to us. We had anticipated that increased ANGPTL3/8 activity would impair the conformation of LPL’s catalytic domain, such that the decrease in LPL activity in the postheparin plasma would be greater than the decrease in LPL mass; however, we found no evidence that this was the case. APOA5 deficiency appeared to reduce the amounts of LPL within capillaries without resulting in a readily detectable effect on LPL specific activity. We suspect that increased ANGPTL3/8 activity in the setting of APOA5 deficiency disrupts LPL conformation, simultaneously triggering enzyme inactivation and rapid detachment of LPL from its intravascular biding sites. Our findings suggest that LPL, after interacting with ANGPTL3/8, does not linger within capillaries and thus does not contribute in a substantial way to the pool of LPL that is released by heparin.

Our findings in *Apoa5^–/–^* mice are clinically relevant. Humans with biallelic *APOA5* loss-of-function mutations have severe hypertriglyceridemia (6, 8, 9), and *APOA5* mutations increase the risk of coronary heart disease (4, 10, 11). Of note, a common *APOA5* missense mutation in Chinese populations (with an allelic frequency of 7%) causes both severe hypertriglyceridemia (7) and increases the risk of coronary heart disease (11). Our current studies suggest that APOA5-deficient patients could be treated effectively with either APOA5 or an inhibitory ANGPTL3/8-specific mAb, but we suspect the long half-life of mAbs could make them the preferred treatment. The mAb that we used, IBA490, contains the Fc region of mouse IgG and the Fab region of a human ANGPTL3/8-specific mAb (24). In a double-blind study of 48 patients with mixed hyperlipidemia, the human ANGPTL3/8 mAb reduced plasma TG levels by 70%, remnant cholesterol levels by 61%, LDL cholesterol levels by 36%, and APOB levels by 31%, while increasing HDL cholesterol levels by 26% (47). After a single dose, the lowering of TG levels persisted for 2 weeks. We suspect that the ANGPTL3/8 mAb, by increasing intracapillary LPL levels, will be highly effective in treating hypertriglyceridemia in patients with APOA5 deficiency. Also, the fact that the ANGPTL3/8 Ab reduced LDL cholesterol and APOB levels in patients strongly suggests that it could prove to be effective in preventing coronary heart disease.

## Methods

### Genetically modified mice.

*Apoa5^–/–^* mice (FVB/NJ) were obtained from the Mutant Mouse Resource and Research Center (MMRRC) at UC Davis. *Gpihbp1^–/–^* (48), *Lpl^–/–^* Tie2–hLPL (22, 49), and *Lpl^+/–^* (23, 50) mice have been described previously. Twelve- to 14-week-old mice were maintained in a barrier facility on a 12-hour light/12-hour dark cycle and were fed a chow diet ad libitum unless otherwise noted. Fasting and refeeding studies were carried out as described previously (28) with minor modifications. Mice were synchronized with 3 cycles of fasting (6:00 pm–9:00 am) and refeeding (9:00 am–6:00 pm). At 9:00 am, after the last cycle, plasma samples were collected, and the mice were refed a chow diet. At 1:00 pm, samples from the refed mice were collected.

### Heparin injections.

Heparin (500 U/kg, McKesson) was administered by intravenous injection. Plasma samples were collected before and 2 minutes after the heparin injection and aliquoted for TG levels, LPL mass and activity levels, and for LPL purification by HS chromatography. Plasma samples were stored at –80°C.

### HS chromatography.

Pooled postheparin plasma (167 μL) was loaded onto a 1.0 mL HS HiTrap column (Cytiva). The column was washed with 10 mL equilibration buffer (0.25 M NaCl, 20% glycerol, 0.01% BSA, 10 mM sodium phosphate, pH 6.5). LPL was eluted with a linear NaCl gradient (0.25–1.5 M NaCl in 20% glycerol, 0.01% BSA, 10 mM sodium phosphate, pH 6.5) (48). Thirty fractions were collected; active LPL appeared in the high-salt fractions (fractions 21–27; 1.13–1.38 M NaCl). LPL mass and activity were determined in each fraction.

### Perfusion of isolated mouse hearts with heparin.

Mice were anesthetized and perfused with 10 mL Tyrode’s buffer via the inferior vena cava (22, 29). The heart was removed and rinsed with Tyrode’s buffer. A blunt needle was inserted into the aorta, clamped in position, and tied down with sutures. The heart was perfused with Tyrode’s buffer (1 mL/min for 3 min). Next, heparin (50 U/mL in Tyrode’s buffer) was injected (1 mL/min for 3 min) to release intravascular LPL. Eight 250 μL fractions were collected and adjusted to 1.2 M NaCl and 50 U/mL heparin.

### Abs and recombinant proteins.

mLPL-specific Abs (rat mAb 27A7, rabbit polyclonal Abs Ab3174 and Ab3175) (23, 29) were raised against mLPL produced in *Drosophila* S2 cells (51). We also used a goat Ab raised against a mLPL fragment produced in *E*. *coli* (30, 52) and a goat Ab against hLPL (Abcam, AF7197) that proved to be useful for detecting mLPL. hLPL was detected with mouse mAb 5D2 (34). GPIHBP1 was detected with rat mAb 11A12 (53). CD31 was detected with the hamster mAb 2H8 (Developmental Studies Hybridoma Bank, University of Iowa, Iowa City, Iowa, USA) (54). Alexa Fluor–labeled secondary Abs were purchased from Thermo Fisher Scientific and Jackson ImmunoResearch, and IRDye-labeled secondary Abs were purchased from LI-COR. IBA490 is an ANGPTL3/8-specific mAb that contains the Fc region of mouse IgG and the Fab region of an inhibitory human ANGPTL3/8 mAb (24). An irrelevant mAb (also containing the Fc region of mouse IgG and the Fab region of a human mAb) was used as a control. Recombinant hLPL was prepared as described previously (33). Mouse APOA5 (HIS–mouse serum albumin–APOA5) and the mouse ANGPTL3/8 complex were produced as described previously (18, 32). IBA490 and the control mAb (10 mg/kg) were administrated subcutaneously, and APOA5 (10 mg/kg) was administered intravenously. Mice were analyzed 4 hours after an injection of APOA5 or 24 hours after the injection of IBA490 (or the control mAb) unless otherwise stated.

### Plasma TG levels and LPL mass and activity levels.

Plasma TG levels were measured with a Serum Triglyceride Determination Kit (MilliporeSigma). Levels of TG hydrolase activity in plasma were determined as described previously (29, 55, 56). Briefly, preheparin and postheparin plasma samples were adjusted to 1.2 M NaCl and 50 U/mL heparin (29). Serial 1:2 dilutions of 15 μL plasma samples were incubated with 6 mM [^3^H]triolein in a Tris buffer (0.15 M Tris, 6% BSA, and 17.9 U/mL heparin, pH 8.5, with a final NaCl concentration of 0.13 M). Heat-inactivated rat serum (5 μL) was used as a source of APOC2. The reaction was stopped by adding 50 μL 10% Triton X-100, 0.5 mL ddH_2_O, and 2 mL isopropanol/heptane/H_2_SO_4_ (800:966:40, v/v/v). After mixing and centrifugation, lipids in the upper phase (0.8 mL) were extracted with 1 mL alkaline ethanol (95% ethanol/ddH_2_O/2 M NaOH, 500:450:50, v/v/v) and 3 mL heptane. After mixing and centrifugation, the upper heptane phase was discarded, and a second heptane wash was performed. The fatty acid products of TG hydrolysis were counted in a LS6500 Scintillation Counter (Beckman Coulter) by mixing 800 μL of the alkaline ethanol phase with 4 mL of a liquid scintillation cocktail (Optiphase, PerkinElmer). TG hydrolase activity was calculated from dilutions falling within the linear range of the curve. TG hydrolase activity of 1.0 mU corresponds to 1 nmol fatty acid release/min. To quantify the TG hydrolase activity due to LPL, we used the method developed and validated by Dallinga-Thie and coworkers (55). With this method, LPL activity is quantified by subtracting the TG hydrolase activity in the preheparin plasma (reflecting hepatic lipase activity) from the TG hydrolase activity in the postheparin plasma (55). We also measured TG hydrolase activity in heart perfusate fractions and HS fractions.

LPL mass was measured with a sandwich ELISA (29). Wells of 96-well plates (Costar) were coated with Ab3175 (0.5 μg/well) overnight at 4°C, washed with 5 U/mL heparin, 0.1% BSA, PBS/Ca/Mg, and blocked for 4 hours at room temperature with StartingBlock buffer (Thermo Fisher Scientific). Next, 1:2 serial dilutions of the standard (0–50 ng/mL recombinant mLPL) (51) and samples (in StartingBlock buffer containing 10 U/mL heparin) were added to wells and incubated overnight at 4°C. The wells were washed, incubated with HRP-27A7 (100 ng/well) for 2 hours at room temperature, and then washed again. One-step Ultra TMB substrate (50 μL/well, Thermo Fisher Scientific) was added to the wells to detect HRP activity, and the reaction was stopped with 2 M sulfuric acid (50 μL/well). OD was recorded at 450 nm with a SpectraMax iD3 plate reader (Molecular Devices). LPL mass was calculated by linear regression from dilutions falling within the linear range of the standard curve. In our studies, the impact of hepatic lipase (HL) activity on TG hydrolase activity measurements was minimal because the TG hydrolase activity assays were performed under conditions (1.2 M NaCl) in which HL activity was inhibited.

### Immunoprecipitation studies and Western blot analyses.

mLPL was immunoprecipitated from 30 μL preheparin plasma with magnetic beads coated with 8 μg goat Ab against mLPL. After washing the beads 3 times with a washing buffer (0.1 M sodium phosphate containing 0.2% NP40, pH 8.0) supplemented with cOmplete protease inhibitor cocktail (1 tablet/5 mL, MilliporeSigma), LPL was eluted with 0.1 M glycine, pH 2.7. For Western blots, proteins were size-fractioned on 4%– 12% Bis-Tris SDS-polyacrylamide gels (Novex, Thermo Fisher Scientific) and transferred to nitrocellulose. LPL was detected with IRDye 800-27A7, IRDye 680-3174, or a goat Ab against mLPL (4 μg/mL) followed by IRDye 680 donkey anti–goat IgG. Ab binding was quantified with an Odyssey infrared scanner (LI-COR).

### Quantitative RT-PCR.

Mouse tissues were collected, snap-frozen, and stored at –80°C. RNA was extracted with TRI reagent (Molecular Research), and cDNA was prepared with random primers, oligo(dT), and SuperScript III (Invitrogen, Thermo Fisher Scientific). Quantitative reverse transcription PCR (RT-PCR) was performed on triplicate samples using SYBR Green PCR Master Mix (Bioland) on a QuantStudio 5 Real-Time PCR System (Applied Biosystems). Transcript levels were measured with the comparative Ct method and normalized to *Ppia* (57).

### Immunohistochemistry.

To detect LPL, GPIHBP1, and CD31 levels in mouse tissues, frozen sections (10 μm thick) were fixed in ice-cold methanol, blocked in 0.2% BSA and 5% donkey serum for 1 hour at room temperature, and then incubated with primary Abs (5 μg/mL 11A12, 5 μg/mL Ab3174, 20 mg/mL 2H8) overnight at 4°C. After washing, sections were incubated with fluorescent secondary Abs (Alexa Fluor 488 anti–hamster IgG, Alexa Fluor 555 anti–rabbit IgG, and Alexa Fluor 647 anti–rat IgG, all at 10 μg/mL) for 1 hour at room temperature. After washing, the sections were stained with DAPI and then rinsed and mounted. Images were acquired with an LSM 980 microscope (Zeiss) with a ×20 or ×63 objective. Three confocal images per tissue, genotype, and treatment were recorded. Fluorescent signals on capillaries were analyzed in Fiji (ImageJ, NIH), with 8 bit original data exported from Zen Blue (Zeiss).

### Assessment of intravascular levels of LPL.

To assess intravascular levels of LPL, GPIHBP1, and CD31, mice were given an intravenous injection of 100 µg Alexa Fluor 488-11A12, 100 µg Alexa Fluor 555-27A7 (or 120 µg Alexa Fluor 555-Ab 3174), and 200 µg Alexa Fluor 647-2H8. After 10 minutes, the vasculature was perfused with PBS followed by 4% PFA. Tissues were collected, fixed in 4% PFA overnight, embedded in OCT, sectioned at 10 μm thickness, mounted onto slides, and examined with an LSM 980 confocal microscope (Zeiss). To assess the binding of Abs in larger tissue sections and with greater accuracy, mice were given an intravenous injection of IRDye 680-27A7 and IRDye 800-11A12 (100 μg each), and tissue sections were examined with an infrared scanner (LI-COR). In similar studies, mice were given an intravenous injection of IRDye 680-11A12 and 120 μg IRDye 800-2H8 (100 μg each).

### Native PAGE.

The ability of 27A7 to bind mLPL•hGPIHBP1 complexes was examined by native gel electrophoresis (40). In brief, 1 μg mLPL or 1 μg mLPL•hGPIHBP1 complexes were preincubated with or without 27A7 (1.7 μg) on ice for 10 minutes. The mixtures were loaded onto 4%–16% native polyacrylamide gels (Novex, Thermo Fisher Scientific) and subjected to a field gradient of 100 V for 10 minutes, 200 V for 30 minutes, and 300 V for 20 minutes at 4°C in a Tris-glycine buffer (pH 8.4). To visualize protein migration, the gels were stained with Coomassie G-250.

### Assessment of the ability of recombinant ANGPTL3/8 to release LPL from the surface of cultured cells.

CHO-K1 cells (American Type Culture Collection [ATCC] CCL-61), which express HSPGs on the cell surface (31), were maintained in F12 medium (Lonza) containing 10% FBS (Gemini) and 1% l-glutamine (Gibco, Thermo Fisher Scientific). After plating the cells on coverslips in 24-well plates, the cells were washed 3 times in PBS/Ca/Mg and then incubated with 50 nM hLPL in F12 medium at 37°C. After 10 minutes, the cells were washed with PBS/Ca/Mg and incubated in medium containing heparin (0.1 U/mL) or were incubated with ANGPTL3/8 (100 nM) in the presence or absence of IBA490 (1 μM), control mAb (1 μM), or recombinant APOA5 (1.4 μM) at 37°C for 15 minutes. After washing the cells, the amounts of hLPL remaining on the cell surface were assessed by immunocytochemistry. The cells were washed 3 times in PBS/Ca/Mg buffer and blocked in 10% donkey serum in PBS/Ca/Mg at 4°C for 1 hour. The cells were then stained with Alexa Fluor 555-5D2 (5 μg/mL) in 3% donkey serum/PBS/Ca/Mg at 4°C for 1 hour. After washing the cells 3 times in 3% donkey serum/PBS/Ca/Mg and 2 times with PBS/Ca/Mg, the cells were fixed, stained with DAPI, rinsed, and mounted. Images were recorded with an LSM 980 microscope (Zeiss) with a ×20 objective. The fluorescence intensity of 5D2 was quantified on the surface of CHO-K1 cells (*n* = 21–62 cells/group).

We also cultured CHO PgsA-745 cells lacking hamster LPL expression (58), transfected the cells with a mGPIHBP1 expression vector (35), and incubated the cells with recombinant hLPL. We then examined the effect of heparin and ANGPTL3/8 (in the presence or absence of IBA490 and APOA5) on LPL binding to GPIHBP1 on the surface of the cells. Our procedures mirrored those described for the CHO-K1 studies. The amount of LPL (relative to GPIHBP1) on the cell surface was assessed by incubating the cells with 5D2 and 11A12 (each labeled with an Alexa Fluor dye). Images were recorded with the LSM 980 microscope. The fluorescence intensity of 5D2, relative to 11A12, was quantified in 90 to 192 cells per experimental group. We also tested the ability of ANGPTL3/8 (in the presence or absence of IBA490 and APOA5) to release LPL from the surface of GPIHBP1-expressing rat heart microvascular endothelial cells (52, 59).

We also tested the ability of ANGPTL3/8 to release mLPL from HEK293 cells that were stably transfected with a mLPL expression vector (NP_032535.2) (18). The cells were maintained in DMEM/F12 (3:1) (Invitrogen, Thermo Fisher Scientific) containing 10% FBS (Hyclone) and 5 μg/mL blasticidin (Invitrogen, Thermo Fisher Scientific). Cells were seeded onto poly-d-lysine 48-well plates (Corning) (160,000 cells/well). After an overnight incubation, the cells were washed with medium DMEM/F12 (3:1) containing 0.1% fatty acid–free BSA (MilliporeSigma) and incubated in medium for an additional 30 minutes at 37°C. The medium was replaced with 200 μL medium containing mouse ANGPTL3/8 (3–300 nM) or heparin (0.1 U/mL) that had been preincubated for 30 minutes at 37°C in the absence or presence of 1 μM mouse APOA5 or 1 μM IBA490. The cells were incubated for 15 minutes, after which the medium was recovered. One-half of the sample was used to measure LPL catalytic activity (32), and the other half was used to assess LPL levels by Western blotting (60). Proteins in the medium were size-fractionated on 4%–12% Bis-Tris polyacrylamide gels and transferred onto PVDF membranes. The membranes were incubated with a goat LPL Ab (AF7197, Abcam) and then an IRDye 680 anti–goat IgG secondary Ab. Images were recorded with a LI-COR Odyssey infrared scanner, and LPL band intensities were quantified using Empiria Studio (LI-COR).

### Statistics.

 Statistical analyses were performed with GraphPad Prism 9.0 (GraphPad Software). A 2-tailed Student’s *t* test was used to compare 2 independent groups. For multiple group comparisons, a 1- or 2-way ANOVA was used. All experiments were repeated 2 or more times, and representative data are shown as the mean ± SD or the mean ± SEM. A *P* value of less than 0.05 was considered statistically significant.

### Study approval.

All studies were approved by the Animal Research Committee of UCLA.

### Data availability.

Values for all data points in the graphs are reported in the Supplemental Supporting Data Values file.

## Author contributions

YY, SGY, APB, and LGF designed the experiments and wrote the manuscript. YY, APB, HJ, YT, LPN, WS, TAW, YQC, and EYZ performed experiments, collected data, and assembled figures. CMT, KX, and APT analyzed data. SGY, LGF, APB, MP, WS, and PT secured funding. YY, APB, MP, KM, KN, MM, YQC, GB, PT, RJK, YT, JK, PHK, and RGY assisted with resources and/or provided expertise and feedback.

## Supplementary Material

Supplemental data

Supporting data values

## Figures and Tables

**Table 1:**
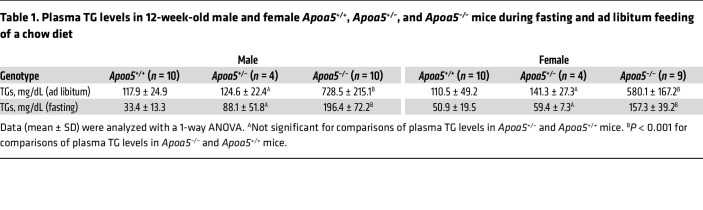
Plasma TG levels in 12-week-old male and female *Apoa5^+/+^*, *Apoa5^+/–^*, and *Apoa5^–/–^* mice during fasting and ad libitum feeding of a chow diet

## Version 1 10/12/2023

In-Press Preview

## Version 2 12/01/2023

Electronic publication
