# Supplementary material for: Hypertriglyceridemia in Apoa5–/– mice results from reduced amounts of lipoprotein lipase in the capillary lumen
Source: J Clin Invest. 2023 Dec 1;133(23):e172600. doi: 10.1172/JCI172600 (PMC10688983; doi:10.1172/JCI172600)
Supplement: Supplemental data [file jci-133-172600-s084.pdf]

Supplemental Figures for:

Hypertriglyceridemia in *Apoa5*$^{-/-}$ mice results from reduced amounts of lipoprotein lipase in the capillary lumen

Ye Yang[1,2], Anne P. Beigneux[1], Wenxin Song[1], Le Phuong Nguyen[1], Hyesoo Jung[1], Yiping Tu[1], Thomas A. Weston[1], Caitlyn M. Tran[1], Katherine Xie[1], Rachel G. Yu[1], Anh P. Tran[1], Kazuya Miyashita[3], Katsuyuki Nakajima[3], Masami Murakami[3], Yan Q. Chen[4], Eugene Y. Zhen[4], Joonyoung Kim[1], Paul H. Kim[1], Gabriel Birrane[5], Peter Tontonoz[6], Michael Ploug[7,8], Robert J. Konrad[4], Loren G. Fong[1,†], and Stephen G. Young[1,2†]

[1]Department of Medicine and [2]Human Genetics, David Geffen School of Medicine, UCLA, Los Angeles, California, USA. [3]Department of Clinical Laboratory Medicine, Gunma University, Graduate School of Medicine, Maebashi, Gunma, Japan; [4]Lilly Research Laboratories, Eli Lilly and Company, Indianapolis, IN, USA; [5]Division of Experimental Medicine, Beth Israel Deaconess Medical Center, Boston, Massachusetts 02215; [6]Department of Pathology and Laboratory Medicine, UCLA, Los Angeles, California, USA; [7]Finsen Laboratory, Copenhagen University Hospital–Rigshospitalet, Copenhagen, Denmark; [8]Biotech Research and Innovation Centre (BRIC), University of Copenhagen, Copenhagen, Denmark

Figures and Figure Legends

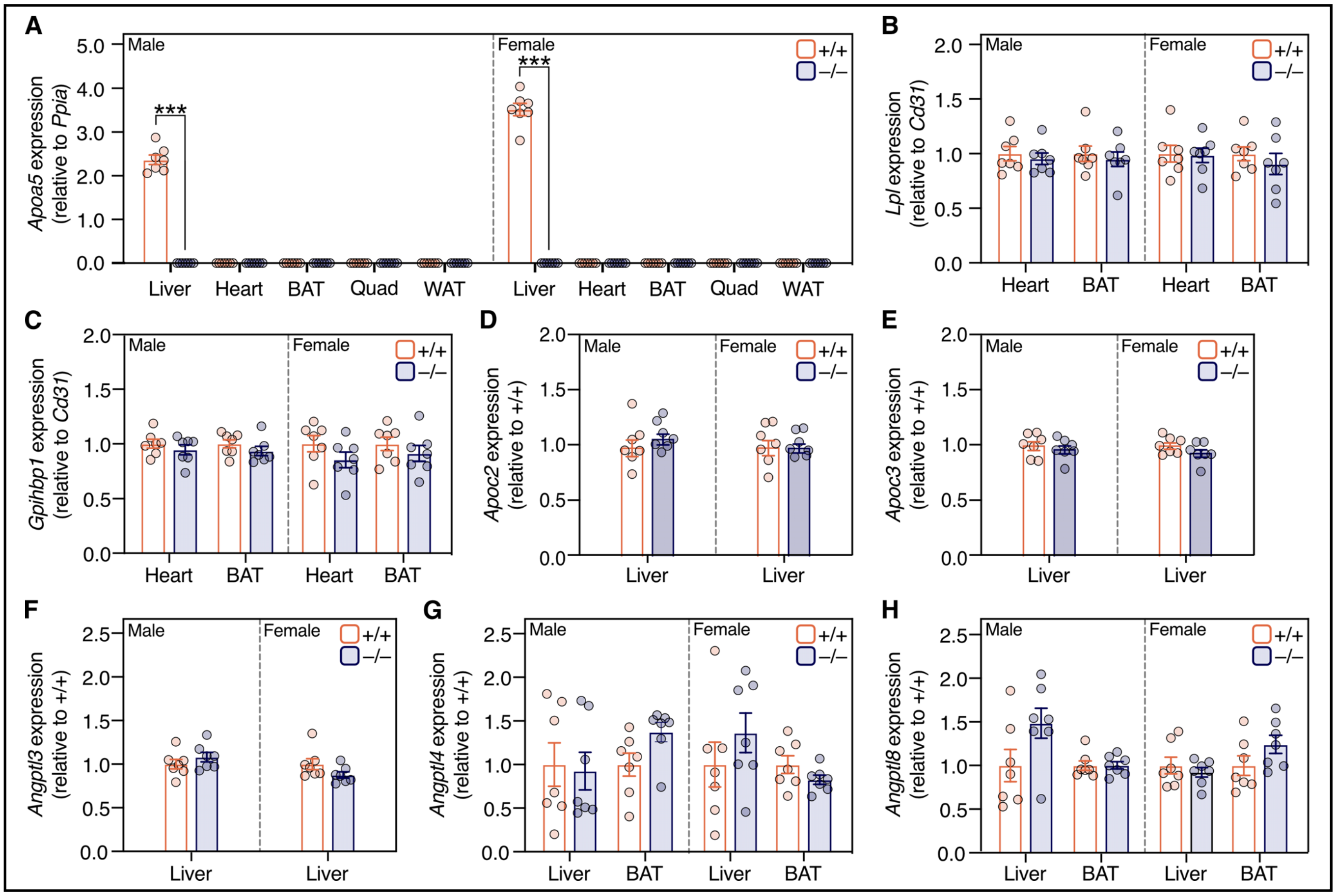

Supplemental Figure 1. Gene expression in *Apoa5*$^{-/-}$ and *Apoa5*$^{+/+}$ mice. (**A**) *Apoa5* transcript levels in the liver, heart, brown adipose tissue (BAT), quadriceps (Quad), and gonadal white adipose tissue (WAT). Each dot represents a single mouse. (**B** and **C**) Transcript levels for *Lpl* (**B**) and *Gpihbp1* (**C**), relative to *Cd31*, in the heart and BAT. (**D–F**) Transcript levels in the liver for *Apoc2* (**D**), *Apoc3* (**E**), and *Angptl3* (**F**). (**G** and **H**) Transcript levels for *Angptl4* (**G**) and *Angptl8* (**H**) in the liver and BAT. In panels **B–H**, transcript levels in *Apoa5*$^{-/-}$ mice were normalized to the mean level in *Apoa5*$^{+/+}$ mice (set at 1.0). Data (mean ± SEM; n = 7/group) were analyzed with an unpaired two-tailed Student's t test. ***$P < 0.001$. In **B–H**, transcript levels between groups were not significantly different.

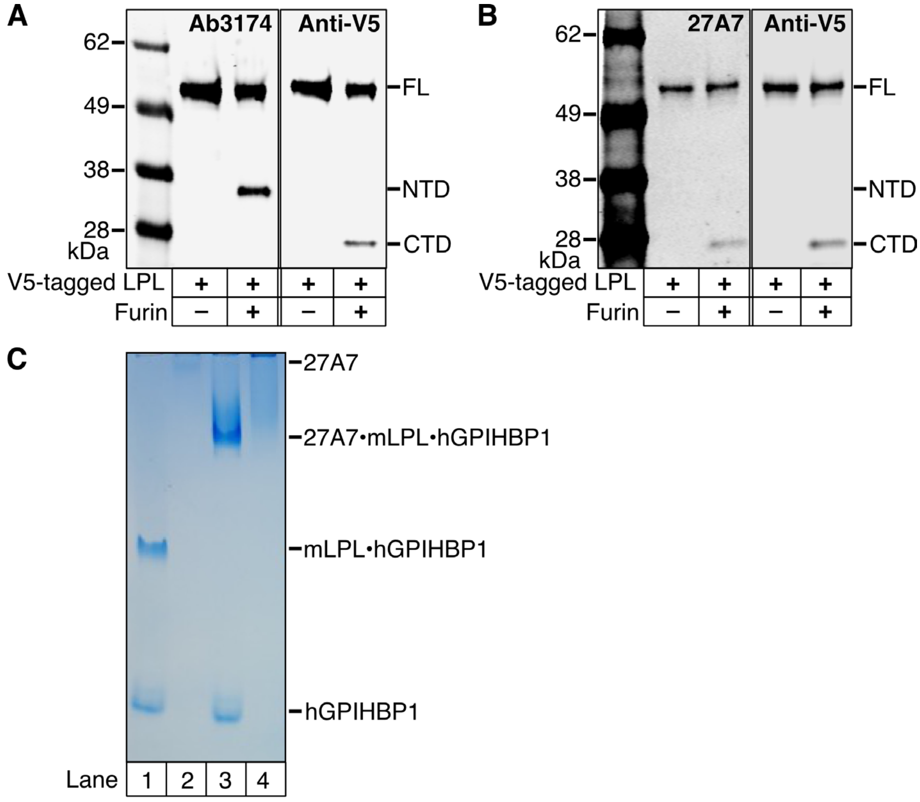

Supplemental Figure 2. Properties of mLPL-specific antibodies. (A–B) Binding of Ab3174 and 27A7 to mLPL that contained a C-terminal V5 tag, incubated in the presence or absence of furin. Furin cleaves full-length (FL) mLPL into an N-terminal catalytic domain fragment (NTD) and a C-terminal lipid-binding fragment (CTD). (**A**) Ab3174 detects FL-LPL and the NTD. (**B**) 27A7 detects FL-LPL and the CTD. (**C**) 27A7 binds to mLPL•GPIHBP1 complexes, as judged by Coomassie blue staining of nondenaturing polyacrylamide gels. Lane 1, mLPL•hGPIHBP1 complexes (1 µg); lane 2, 27A7 (1.7 µg); lane 3, 27A7 (1.7 µg) in the presence of mLPL•hGPIHBP1 complexes (1 µg); lane 4, mAb 27A7 (1.7 µg) in the presence of mLPL (1 µg). LPL enters the gel only when complexed to GPIHBP1.

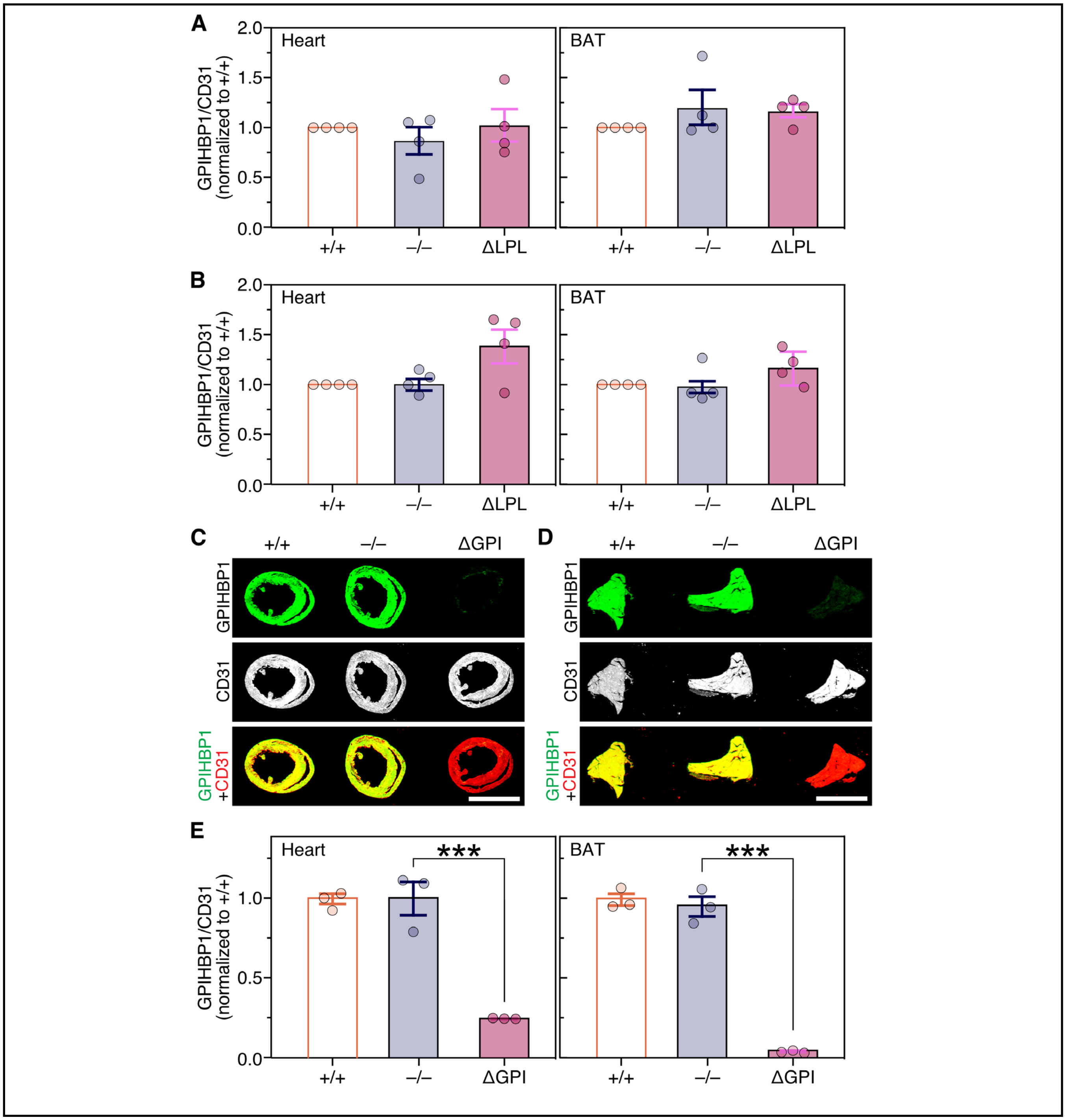

Supplemental Figure 3. Amounts of GPIHBP1, relative to CD31, in heart and BAT capillaries are similar in *Apoa5*$^{-/-}$ and *Apoa5*$^{+/+}$ mice. (**A**) GPIHBP1/CD31 fluorescence intensity ratios in capillaries of *Apoa5*$^{-/-}$, *Apoa5*$^{+/+}$, and *Lpl*$^{-/-}$Tie2–hLPL (ΔLPL) mice after staining tissues with 11A12 and 2H8 (n = 4 independent experiments). (**B**) GPIHBP1/CD31 fluorescent intensity ratios in capillaries of *Apoa5*$^{-/-}$, *Apoa5*$^{+/+}$, and ΔLPL mice after infusions of Alexa Fluor–labeled 11A12 and 2H8 (n = 4 independent experiments). Each dot in panels **A** and **B** represents the GPIHBP1/CD31 signal intensity ratio in one mouse, normalized to the ratio in *Apoa5*$^{+/+}$ mice (set at 1.0). Data (mean ± SEM) were analyzed with a one-way ANOVA test. The GPIHBP1/CD31 ratios in *Apoa5*$^{-/-}$, *Apoa5*$^{+/+}$, and ΔLPL mice were not significantly

different. (**C**–**E**) Amounts of GPIHBP1 and CD31 in sections of the heart or a BAT pad. *Apoa5*$^{+/+}$, *Apoa5*$^{–/–}$, and *Gpihbp1*$^{–/–}$ (ΔGPI) mice were given an intravenous injection of IRDye680-11A12 and IRDye800-2H8. 10 min later, the mice were euthanized; the vasculature was perfused; and tissue sections were prepared for infrared scanning. (**C** and **D**) Infrared scans of heart (**C**) and BAT (**D**). Scale bars, 5 mm. (**E**) GPIHBP1/CD31 ratios in the heart and BAT of *Apoa5*$^{+/+}$, *Apoa5*$^{–/–}$, and ΔGPI mice (n = 3/group). Signal intensities for GPIHBP1 and CD31 were analyzed from ten sections from each mouse. Each dot represents the mean GPIHBP1/CD31 signal intensity ratio in one mouse; data were normalized to the mean ratio in *Apoa5*$^{+/+}$ mice (set at 1.0). Data show mean ± SEM. GPIHBP1/CD31 ratios in *Apoa5*$^{–/–}$ and *Apoa5*$^{+/+}$ mice were not statistically different. As expected, ratios were lower in ΔGPI mice than in *Apoa5*$^{+/+}$ and *Apoa5*$^{–/–}$ mice; ***$P < 0.001$ by a one-way ANOVA test.

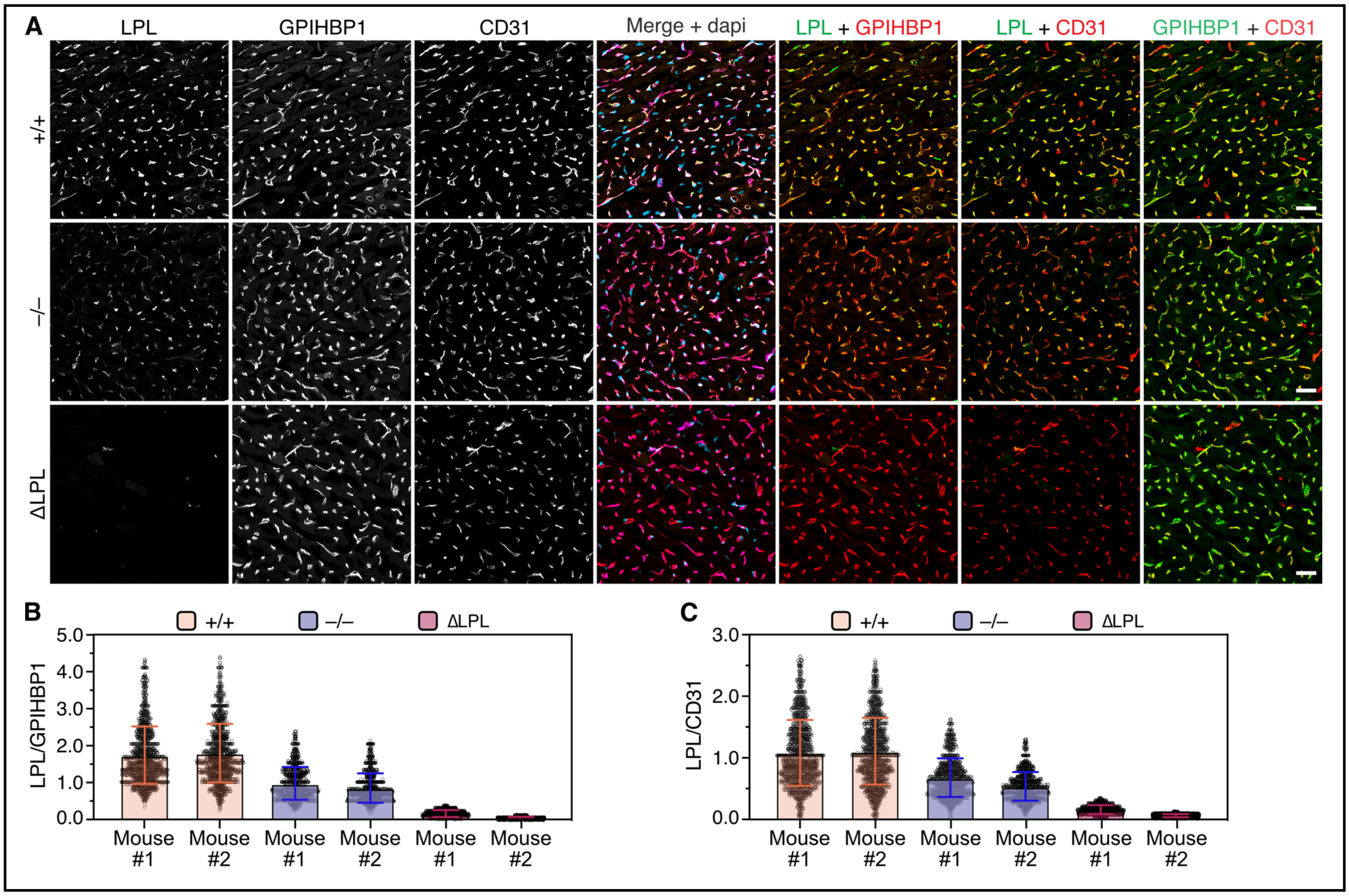

Supplemental Figure 4. Amounts of LPL in heart capillaries, relative to GPIHBP1 or CD31, are lower in *Apoa5*$^{–/–}$ mice. *Apoa5*$^{+/+}$, *Apoa5*$^{–/–}$, and *Lpl*$^{–/–}$Tie2–hLPL (ΔLPL) mice (n = 2/group) were given an intravenous injection of Alexa Fluor–labeled Ab3174 (against mLPL), 11A12 (against GPIHBP1), and 2H8 (against CD31). 10 min later, the mice were perfused with PBS, and tissue sections were prepared for fluorescence microscopy. (**A**) Confocal micrographs showing intracapillary levels of LPL, GPIHBP1, and CD31 in the heart of *Apoa5*$^{+/+}$, *Apoa5*$^{–/–}$, and ΔLPL mice. Scale bars, 20 µm. LPL/GPIHBP1 (**B**) and LPL/CD31 (**C**) fluorescence intensity ratios in heart capillaries of *Apoa5*$^{+/+}$, *Apoa5*$^{–/–}$, and ΔLPL mice. LPL, GPIHBP1, and CD31 fluorescence intensities in individual capillaries were recorded; the number of capillaries examined ranged from 1,228 to 1,606. Each dot represents the ratio in a single capillary. The LPL/GPIHBP1 and LPL/CD31 fluorescence intensity ratios were higher in *Apoa5*$^{+/+}$ mice than in *Apoa5*$^{–/–}$ mice. Data show mean ± SD.

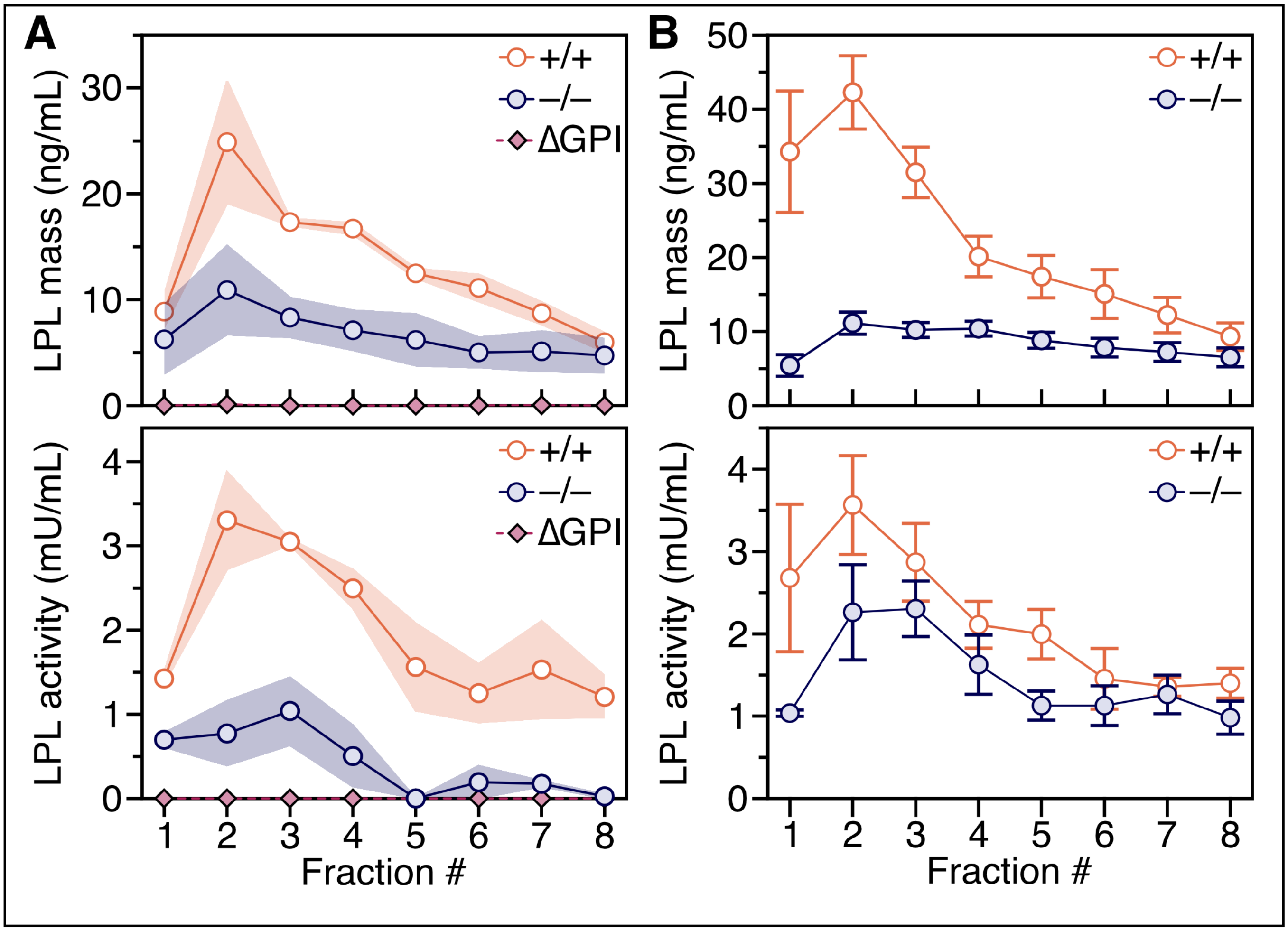

Supplemental Figure 5. The amount of LPL released from isolated perfused mouse hearts after a bolus of heparin is lower in *Apoa5*$^{-/-}$ mice than in *Apoa5*$^{+/+}$ mice. *Apoa5*$^{-/-}$ and *Apoa5*$^{+/+}$ mice were euthanized and perfused with Tyrode's buffer. The heart was removed; a blunt needle was inserted into the aorta; and the coronary circulation was perfused with Tyrode's buffer. Next, 2 mL of Tyrode's buffer containing 50 U/mL heparin was infused (1 mL/min). Perfusates were collected in eight 250-µl fractions. (**A** and **B**) Levels of LPL mass (*top*) and activity (*bottom*) released from isolated hearts of *Apoa5*$^{-/-}$ and *Apoa5*$^{+/+}$ mice in two independent studies. (**A**) *Apoa5*$^{+/+}$ mice, $n = 2$; *Apoa5*$^{-/-}$ mice, $n = 2$; *Gpihbp1*$^{-/-}$ mouse, $n = 1$. Data show mean levels and range. (**B**) *Apoa5*$^{+/+}$ mice, $n = 5$; *Apoa5*$^{-/-}$ mice, $n = 3$. Data show mean ± SEM.

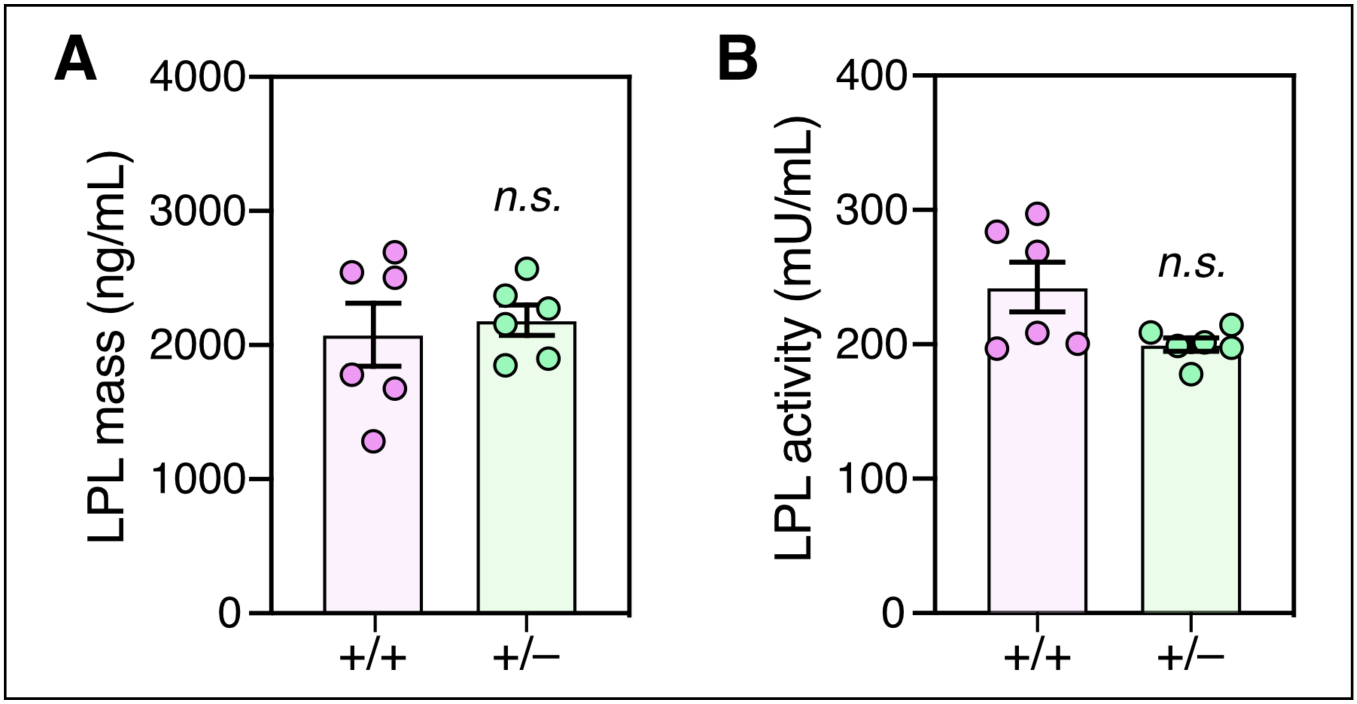

Supplemental Figure 6. Similar levels of LPL mass and activity in the postheparin plasma of *Lpl*$^{+/+}$ and *Lpl*$^{+/–}$ mice. *Lpl*$^{+/–}$ and *Lpl*$^{+/+}$ mice (n = 6/group) were given an intravenous injection of heparin (500 U/kg). After 2 min, plasma was obtained for measurements of LPL mass (**A**) and activity (**B**). Each dot represents the LPL level in the postheparin plasma of a single mouse, normalized to the mean level in *Lpl*$^{+/+}$ mice (set at 1.0). Data (mean ± SEM) were analyzed with a Student's *t* test. NS, not significant.

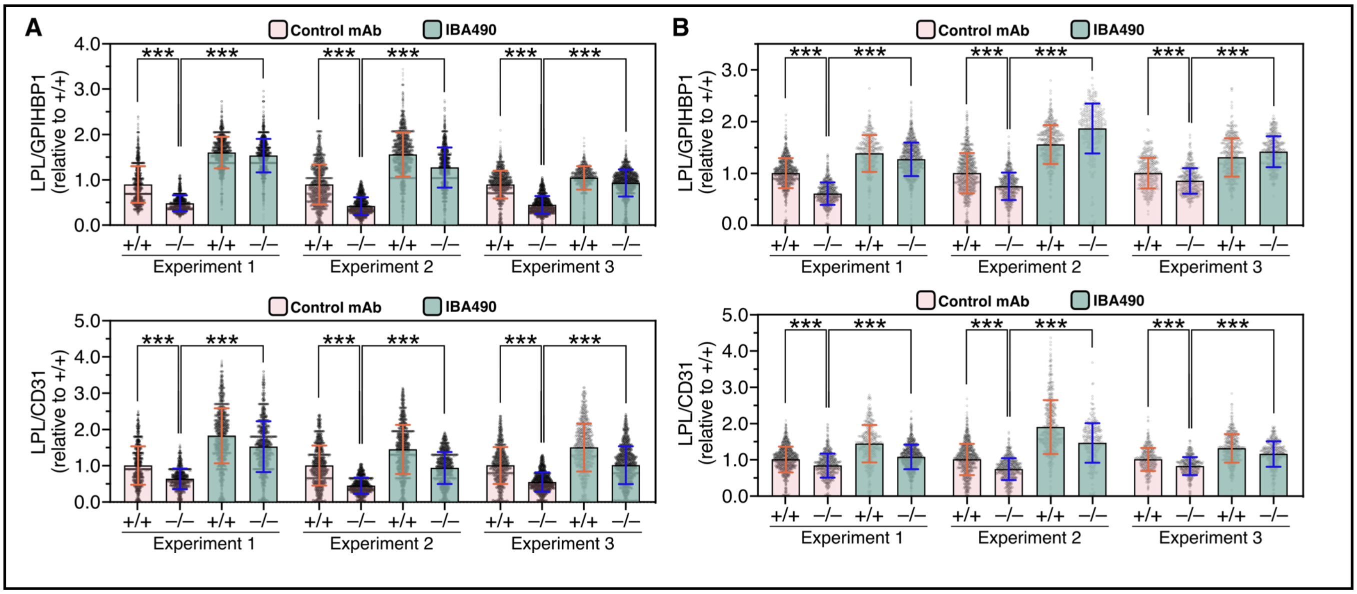

Supplemental Figure 7. IBA490 increases the amount of LPL, relative to GPIHBP1 or CD31, in heart and BAT capillaries of *Apoa5*$^{-/-}$ mice. IBA490- or control mAb–treated *Apoa5*$^{-/-}$ and *Apoa5*$^{+/+}$ mice were given an intravenous injection of Alexa Fluor–labeled 27A7, 11A12, and 2H8. After purfusing the vasculature, tissue sections were prepared for fluorescence microscopy ($n = 3$ independent experiments; 2–4 micrographs/section). LPL, GPIHBP1, and CD31 fluorescence intensities were recorded in individual capillaries; the number of capillaries examined ranged from 381 to 1,970. (**A** and **B**) Bar graphs (mean ± SD) showing LPL/GPIHBP1 and LPL/CD31 fluoresence intensity ratios in capillaries of heart (**A**) and BAT (**B**) of IBA490- or control mAb–treated *Apoa5*$^{-/-}$ and *Apoa5*$^{+/+}$ mice. Each dot represents the signal intensity ratio in a single capillary; data were normalized to the mean ratio in capillaries of control mAb–treated *Apoa5*$^{+/+}$ mice (set as 1.0). ***$P < 0.001$ by a one-way ANOVA test. This figure focuses on differences in fluorescence intensity ratios in *Apoa5*$^{-/-}$ and *Apoa5*$^{+/+}$ mice treated with the control mAb and differences in fluorescence intensity ratios in *Apoa5*$^{-/-}$ mice that were given IBA490 vs. the control mAb. In each experiment, however, we observed higher LPL/GPIHBP1 and LPL/CD31 fluoresence intensity ratios in capillaries of IBA490-treated *Apoa5*$^{+/+}$ mice than in the capillaries of control mAb–treated *Apoa5*$^{+/+}$ mice ($P < 0.001$).

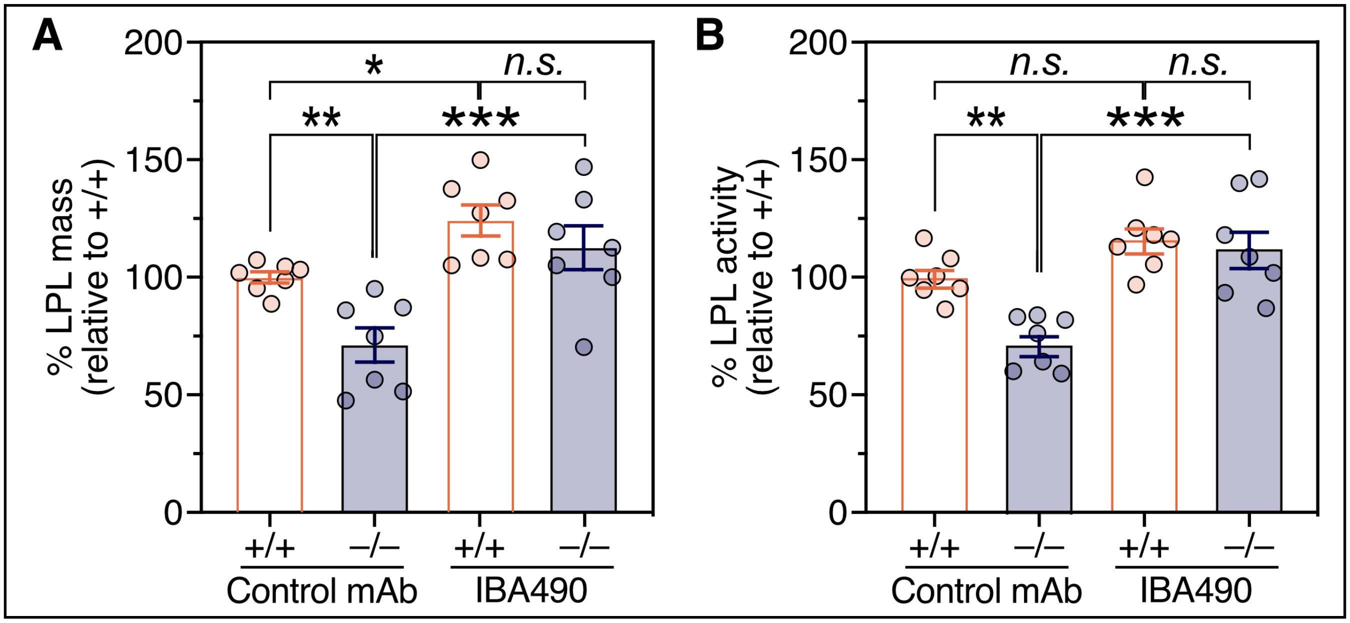

Supplemental Figure 8. Increased amounts of LPL in the postheparin plasma of *Apoa5*$^{-/-}$ mice after an injection of IBA490. *Apoa5*$^{-/-}$ and *Apoa5*$^{+/+}$ mice were given an injection of IBA490 or a control mAb; then, after 4 h, the mice were given an intravenous injection of heparin (500 U/kg). Plasma samples were collected 2 min after the heparin injection. (**A** and **B**) Levels of LPL mass (**A**) and LPL activity (**B**) in the postheparin plasma of IBA490- or control mAb–treated *Apoa5*$^{-/-}$ and *Apoa5*$^{+/+}$ mice. Each dot represents the LPL level in the postheparin plasma of a single mouse, normalized to levels in control mAb–treated *Apoa5*$^{+/+}$ mice (set at 1.0). Data (mean ± SEM) were analyzed with a one-way ANOVA test. NS, not significant. *$P < 0.05$, **$P < 0.01$, ***$P < 0.001$.

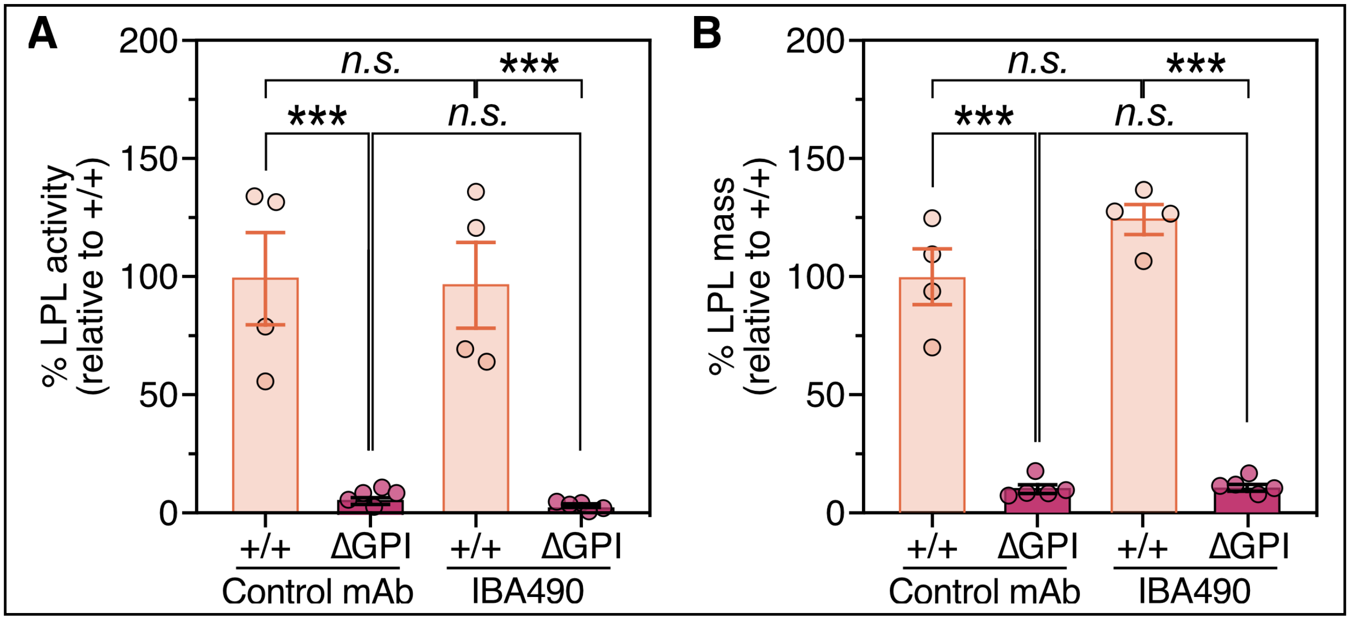

Supplemental Figure 9. IBA490 had no significant effects on LPL activity or LPL mass in the postheparin plasma from *Gpihbp1*$^{-/-}$ mice. *Gpihbp1*$^{-/-}$ and *Gpihbp1*$^{+/+}$ mice were given a subcutaneous injection of IBA490 or a control mAb; then, after 24 h, the mice were given an intravenous injection of heparin (500 U/kg). After 2 min, plasma samples were collected. In *Gpihbp1*$^{-/-}$ mice, the levels of LPL activity (**A**) and LPL mass (**B**) in the postheparin plasma were very low and not significantly affected by IBA490. Each dot represents the LPL level in the postheparin plasma of a single mouse, normalized to levels in control mAb–treated *Gpihbp1*$^{+/+}$ mice (set at 1.0). Data (mean ± SEM) were analyzed with a one-way ANOVA test. NS, not significant. ***$P < 0.001$.

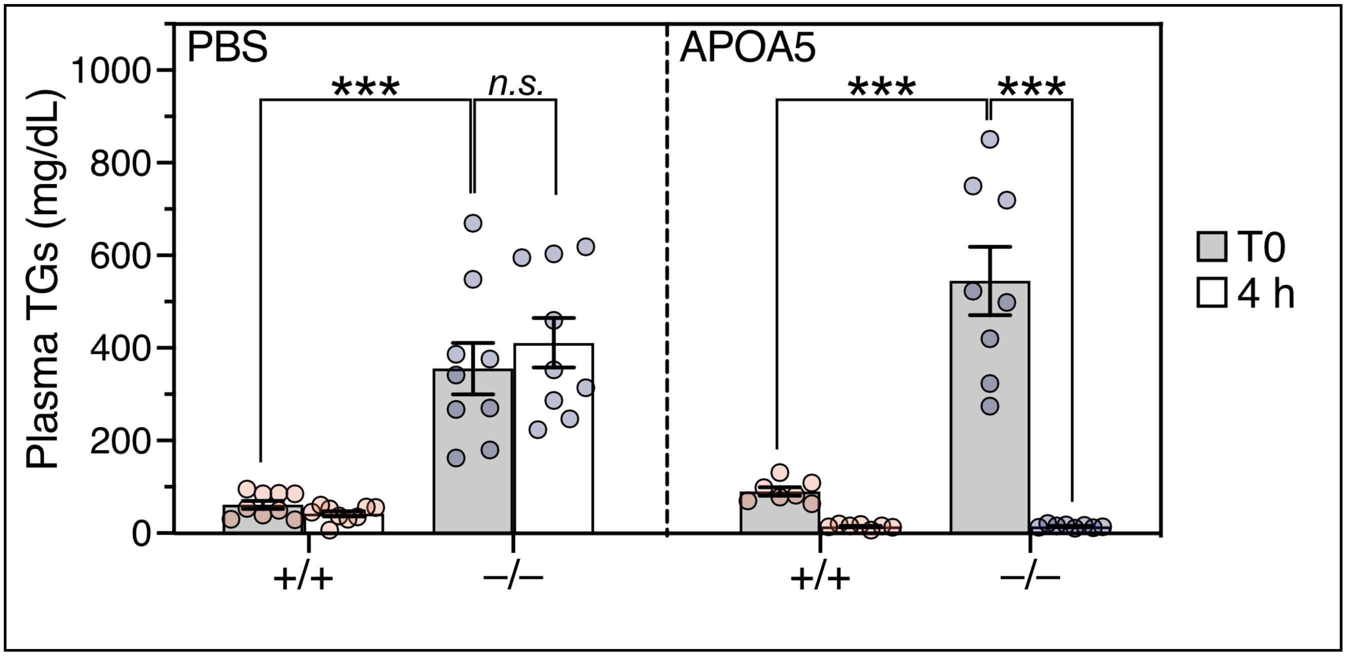

Supplemental Figure 10. Plasma triglyceride (TG) levels in *Apoa5*$^{-/-}$ mice that were treated with recombinant APOA5. *Apoa5*$^{-/-}$ and *Apoa5*$^{+/+}$ mice were given an intravenous injection of APOA5 (10 mg/kg) or PBS alone (n = 7–9/group), and plasma TG levels were measured at baseline (T0) and after 4 h. Data (mean ± SEM) were analyzed with a two-way ANOVA test. NS, not significant. ***$P < 0.001$.

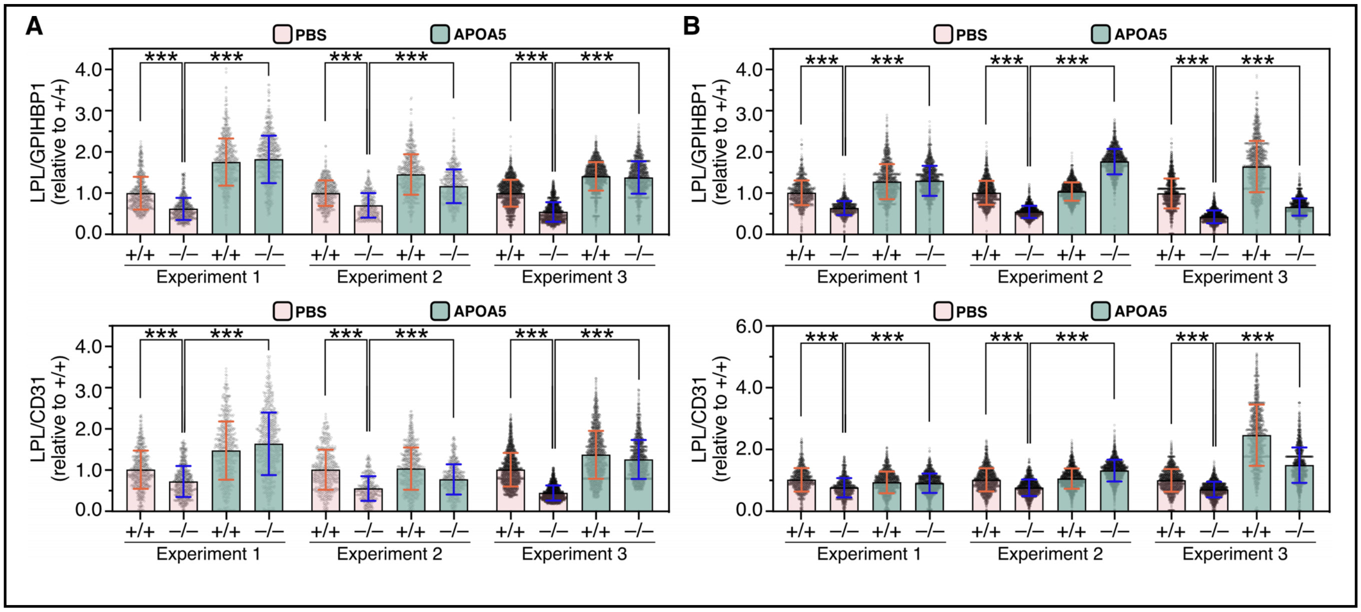

Supplemental Figure 11. Increased amounts of LPL in heart and brown adipose tissue (BAT) capillaries, relative to GPIHBP1 or CD31, in *Apoa5*$^{–/–}$ mice after an intravenous injection of recombinant APOA5. *Apoa5*$^{–/–}$ and *Apoa5*$^{+/+}$ mice were given an injection of recombinant APOA5 (10 mg/kg) or PBS alone. After 4 h, the mice were given an intravenous injection of Alexa Fluor–labeled mAbs against LPL (27A7), GPIHBP1 (11A12), and CD31 (2H8). After perfusing the vasculature with PBS, tissue sections were prepared for fluorescence microscopy (*n* = 3 independent experiments; 2–4 micrographs/tissue section). LPL, GPIHBP1, and CD31 fluorescence intensities were recorded in individual capillaries; the number of capillaries examined ranged from 384 to 2,020. (**A**–**B**) Bar graphs showing LPL/GPIHBP1 and LPL/CD31 fluorescence intensity ratios in capillaries of heart (**A**) and BAT (**B**) of APOA5- or PBS-treated *Apoa5*$^{–/–}$ and *Apoa5*$^{+/+}$ mice. Each dot represents the signal intensity ratio in a single capillary, normalized to the ratio in capillaries of PBS-treated *Apoa5*$^{+/+}$ mice (set as 1.0). Data (mean ± SD) were analyzed with a one-way ANOVA test. ***$P < 0.001$.

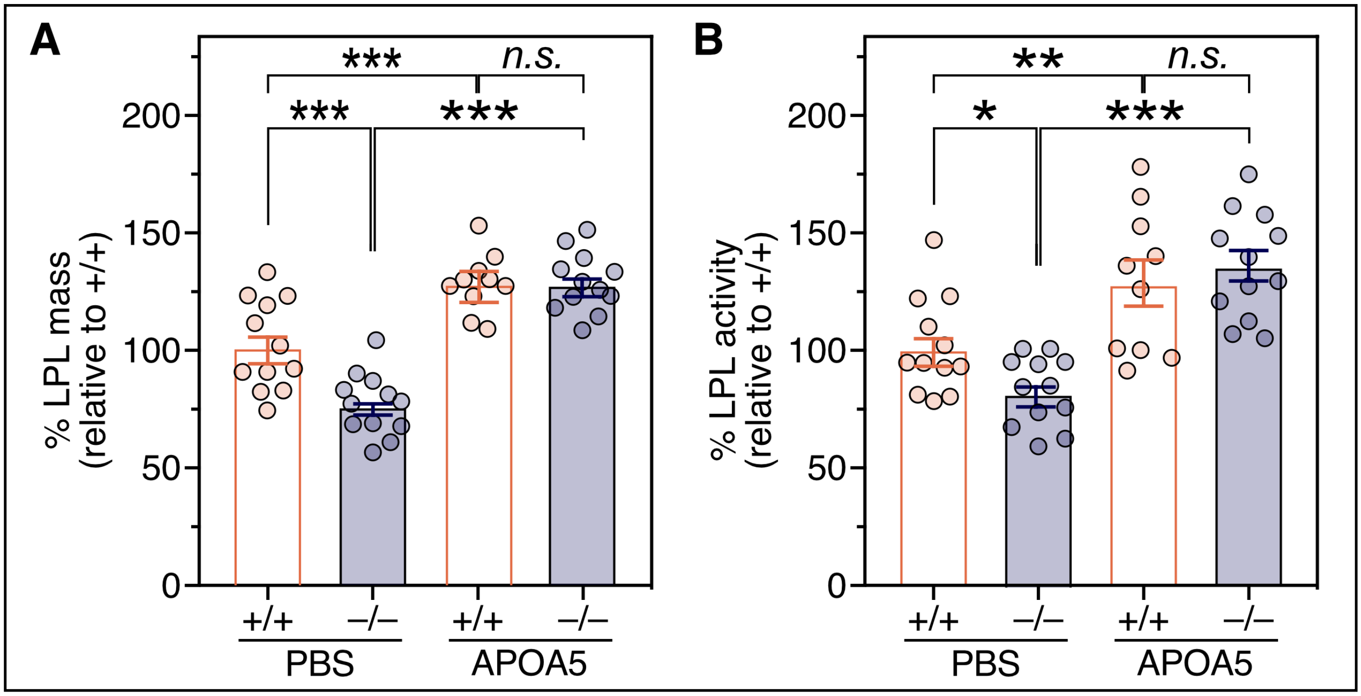

Supplemental Figure 12. Recombinant APOA5 increases LPL levels in the postheparin plasma of *Apoa5*$^{-/-}$ mice. *Apoa5*$^{-/-}$ and *Apoa5*$^{+/+}$ mice were given an intravenous injection of APOA5 (10 mg/kg) or PBS alone. After 4 h, the mice were given an intravenous injection of heparin (500 U/kg). Plasma samples were collected 2 min later. (**A** and **B**) Levels of LPL mass (**A**) and activity (**B**) in the postheparin plasma of *Apoa5*$^{-/-}$ and *Apoa5*$^{+/+}$ mice that had been given injections of APOA5 or PBS. Each dot represents the LPL level in the postheparin plasma of a single mouse, normalized to the mean level in control mAb–treated *Gpihbp1*$^{+/+}$ mice (set at 1.0). Data show mean ± SEM; differences were analyzed with a one-way ANOVA test. NS, not significant. *$P < 0.05$, **$P < 0.01$, ***$P < 0.001$.

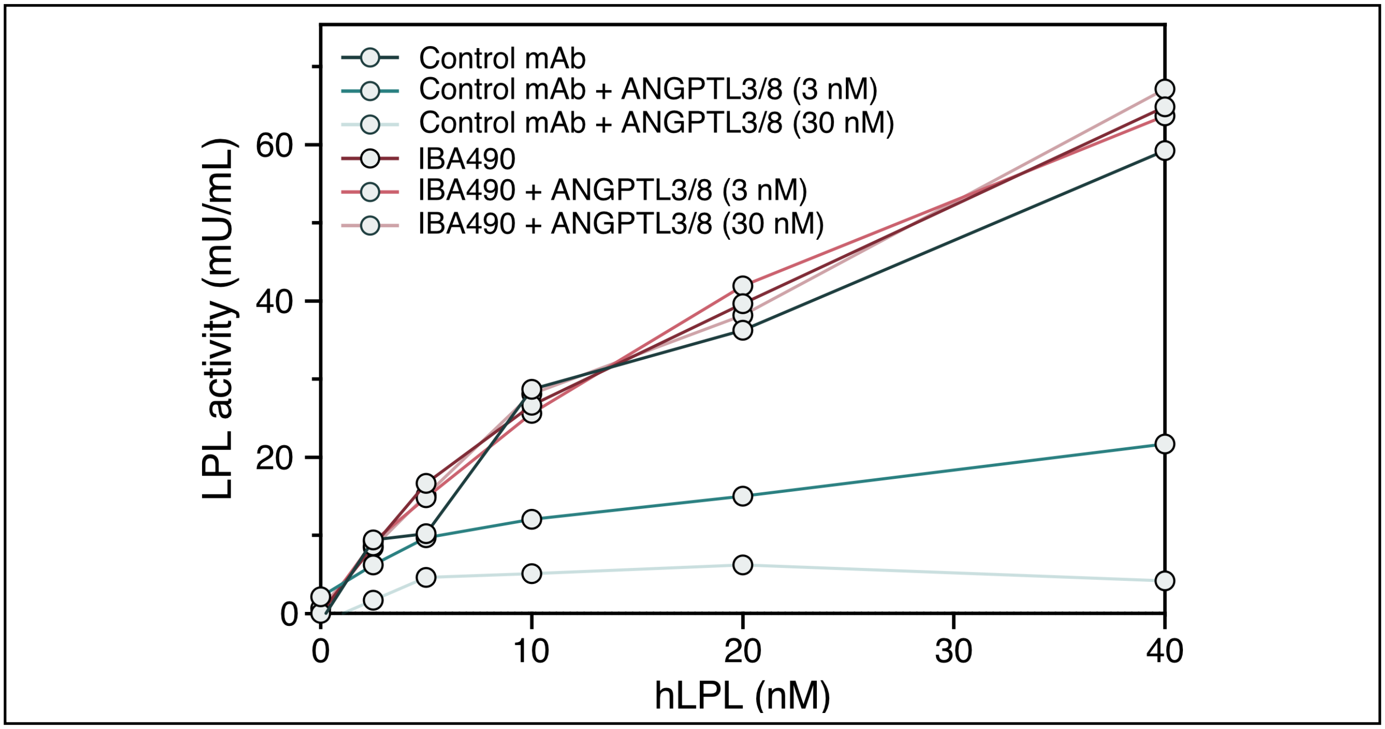

Supplemental Figure 13. IBA490 reduces ANGPTL3/8-mediated inhibition of human LPL (hLPL) activity. ANGPTL3/8 (0–30 nM) was mixed with either IBA490 or a control mAb (1 µM). Then, after 5 min, the ANGPTL3/8–mAb mixtures were added to hLPL (0–40 nM) and incubated for 15 min at room temperature. Triglyceride hydrolase activity was measured with a [^{3}H]triolein substrate.

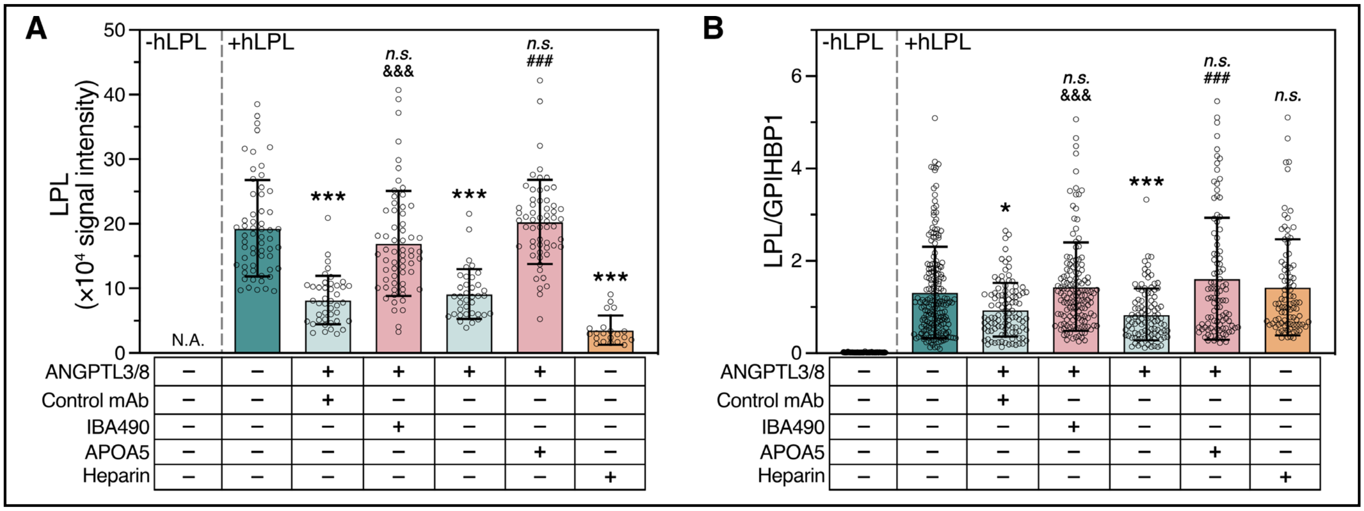

Supplemental Figure 14. ANGPTL3/8 reduces amounts of human LPL (hLPL) on the surface of cells. CHO-K1 cells (**A**) and CHO pgsA-745 cells that had been transiently transfected with a mouse GPIHBP1 expression vector (**B**) were incubated with 50 nM recombinant hLPL for 10 min at 37°C. After washing with PBS/Ca/Mg, the cells were then incubated in serum-free medium containing 0.1 U/mL heparin or were incubated with 100 nM ANGPTL3/8 in the presence or absence of 1 µM control mAb, 1 µM IBA490, or 1.4 µM APOA5. Following a 15-min incubation at 37°C, amounts of hLPL on the surface of cells were assessed by fluorescence microscopy with an Alexa Fluor–labeled mAb against hLPL (5D2) in panel A or with Alexa Fluor–labeled mAbs 5D2 and 11A12 in panel B. (**A**) LPL fluorescence intensity ratios on the surface of CHO-K1 cells. (**B**) LPL/GPIHBP1 fluorescence intensity ratios on the surface of GPIHBP1-expressing CHO pgsA-745 cells. Each dot represents the signal intensity ratio in a single cell. 21–62 cells were analyzed in panel A; 90–192 cells were analyzed in panel B. Data (mean ± SD) were analyzed with a one-way ANOVA test. $*P < 0.05$, $***P < 0.001$, and NS (not significant); comparing heparin- or ANGPTL3/8-treated cells to untreated cells. $^{\&\&\&}P < 0.001$; comparing ANGPTL3/8-treated cells that were incubated in the presence of IBA490 or the control mAb. $^{\#\#\#}P < 0.001$; comparing ANGPTL3/8-treated cells that were incubated in the presence or absence of APOA5.

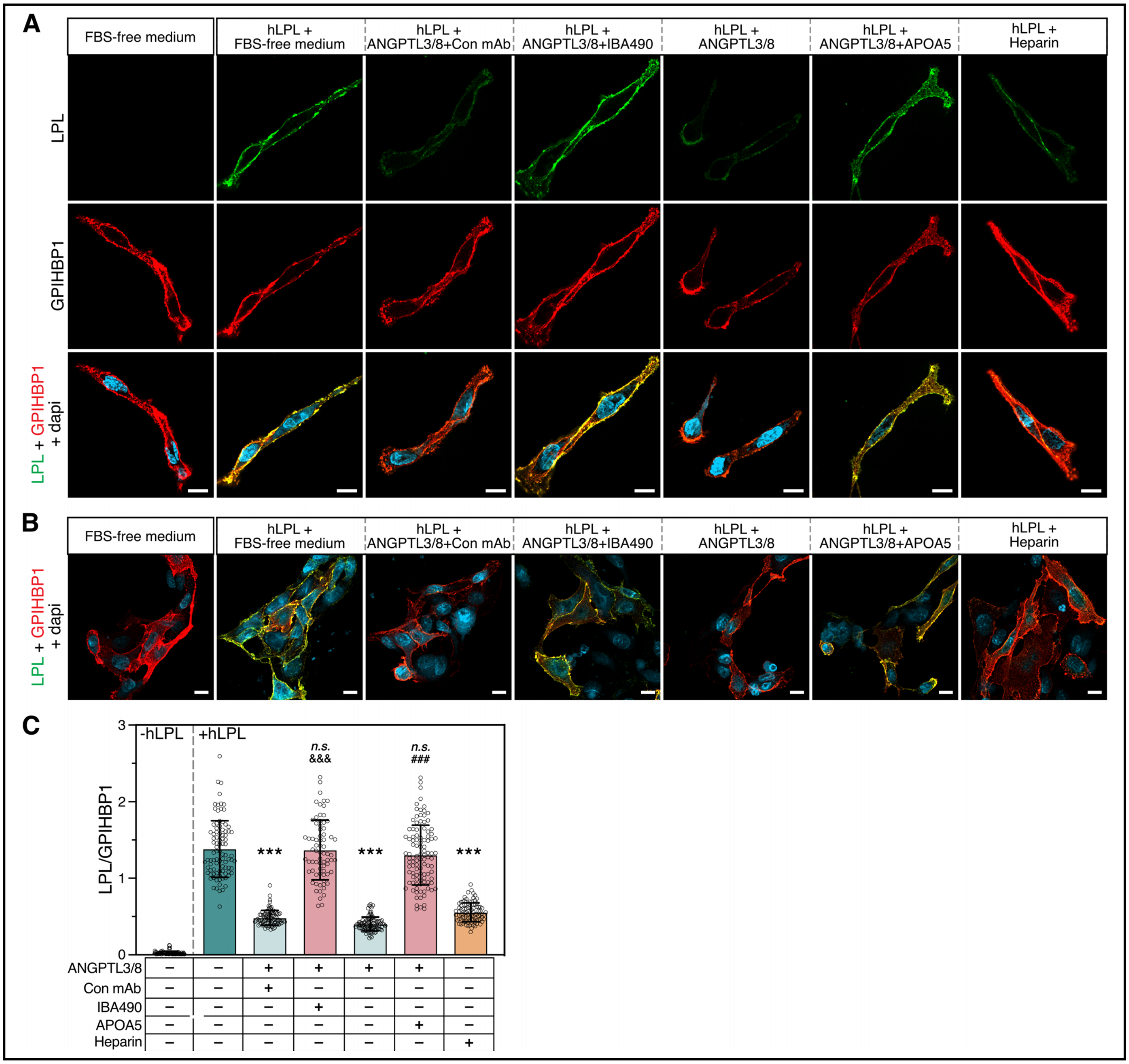

Supplemental Figure 15. ANGPTL3/8 releases hLPL from GPIHBP1-expressing endothelial cells. Primary rat heart microvascular endothelial cells (RHMECs) that had been transduced with a mouse *Gpihbp1* lentiviral vector were incubated with 50 nM recombinant hLPL for 10 min at 37°C. After washing with PBS/Ca/Mg, the cells were then incubated in serum-free medium containing 0.1 U/mL heparin or were incubated with 100 nM ANGPTL3/8 in the presence or absence of 1 µM control mAb, 1 µM IBA490, or 1.4 µM APOA5. Following a 15-min incubation at 37°C, the cells were placed on ice for 15 min. (**A**) Cell surface–hLPL and GPIHBP1 were examined by fluorescence microscopy with Alexa Fluor–labeled mAbs 5D2 and 11A12. (**B**) Amounts of hLPL, relative to GPIHBP1, on the surface of cells in low-magnification images. (**C**) LPL/GPIHBP1 fluorescence intensity ratios on the surface of *Gpihbp1*-expressing RHMECs. Each dot represents the signal intensity ratio in a single cell. 75–107 cells were analyzed. Data (mean ± SD) were analyzed with a one-way ANOVA test. $^{***}P < 0.001$, and NS (not significant); comparing heparin- or ANGPTL3/8-treated cells to untreated cells. $^{\&\&\&}P < 0.001$; comparing ANGPTL3/8-treated cells that

were incubated in the presence of IBA490 or the control mAb. $^{###}P < 0.001$; comparing ANGPTL3/8-treated cells that were incubated in the presence or absence of APOA5.

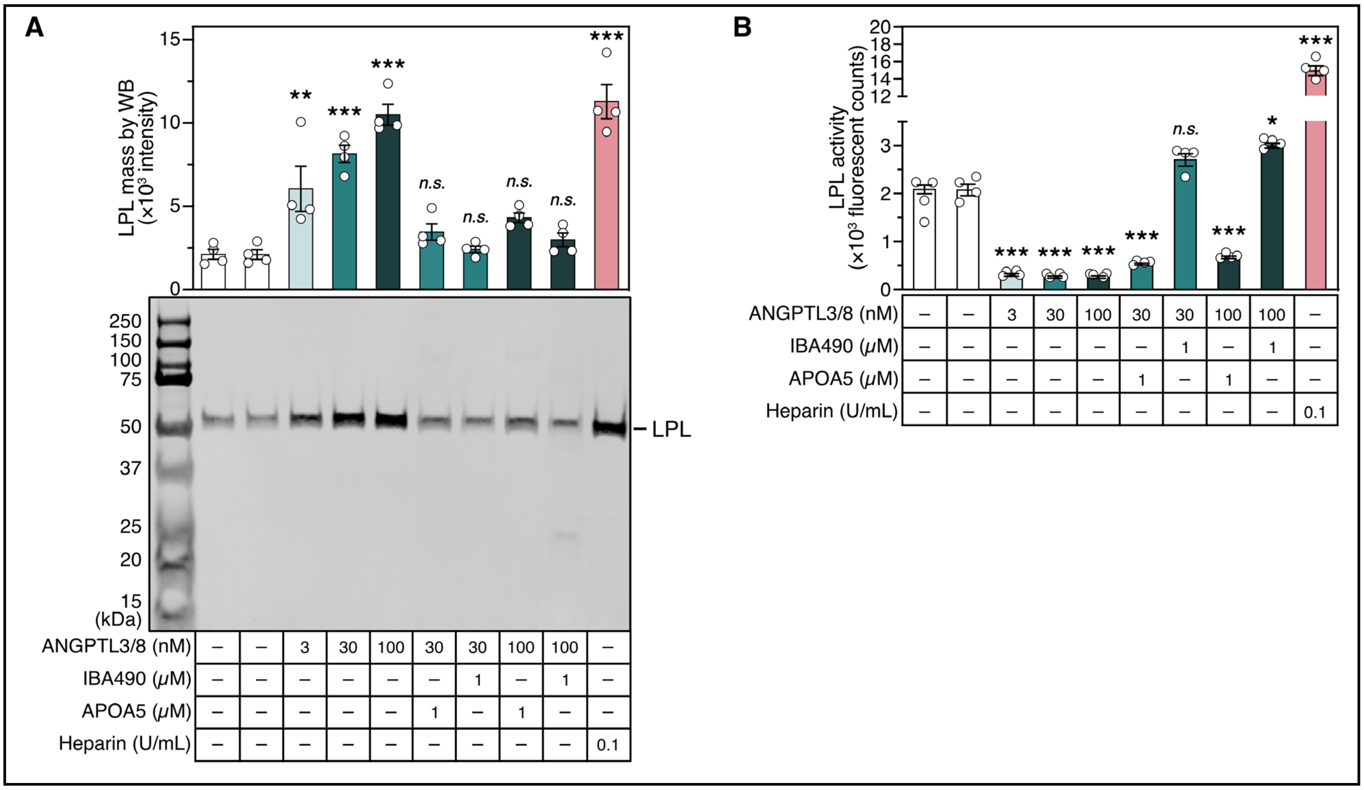

Supplemental Figure 16. ANGPTL3/8 releases mLPL from mLPL-expressing HEK293 cells. HEK293 cells (that are stably expressing mLPL) were incubated with 0.1 U/mL heparin or 0–100 nM ANGPTL3/8 in the presence or absence of 1 µM IBA490 or 1 µM APOA5 at 37ºC for 30 min. (**A**–**B**) Western blot studies (**A**) and LPL activity measurements (**B**) of mLPL in the medium. The dots represent measurements from two independent experiments (two replicates per experiment). Data (mean ± SEM) were analyzed with a one-way ANOVA test. *P < 0.05, **P < 0.01, ***P < 0.001, and NS (not significant); comparing mLPL levels in the culture medium of ANGPTL3/8- or heparin-treated cells and untreated cells.

Supplemental Table. qPCR primer sequence.

**Gene**	**Forward**	**Reverse**
Apoa5	CAGTTGGAGCAAAGGCGTGATG	CTCTCAAGGGTCCCAGCTTTTC
Lpl	AGGTGGACATCGGAGAACTG	TCCCTAGCACAGAAGATGACC
Gpihbp1	AGCAGGGACAGAGCACCTCT	AGACGAGCGTGATGCAGAAG
Cd31	AACCGTATCTCCAAAGCCAGT	CCAGACGACTGGAGGAGAACT
Apoc2	GTTACTGGACCTCTGCCAAGGA	ATGCCTGCGTAAGTGCTCATGG
Angptl3	ACGAAAAGGGCTTTGGGAGGCT	CGTAGTGCTTGCTGTCTTTCCAG
Angptl4	CAAGACCATGACCTCCGTGG	CCGTGGGATAGAGTGGAAGTA
Angptl8	GACTACAAGTGCAGCTGAGAGG	CAGTGAGAGCCCATAAGAGGTG
